# Quantification and Mapping of Alkylation in the Human Genome Reveal Single Nucleotide Resolution Precursors of Mutational Signatures

**DOI:** 10.1021/acscentsci.2c01100

**Published:** 2023-02-22

**Authors:** Yang Jiang, Cécile Mingard, Sabrina M. Huber, Vakil Takhaveev, Maureen McKeague, Seiichiro Kizaki, Mirjam Schneider, Nathalie Ziegler, Vera Hürlimann, Julia Hoeng, Nicolas Sierro, Nikolai V. Ivanov, Shana J. Sturla

**Affiliations:** † Department of Health Sciences and Technology, ETH Zurich, Schmelzbergstrasse 9, Zurich 8092, Switzerland; ‡ Pharmacology and Therapeutics, Chemistry, McGill University, 801 Sherbrooke Street West, Montreal, Quebec H3A 0B8, Canada; § Philip Morris Products SA, Quai Jeanrenaud 3, Neuchatel 2000, Switzerland

## Abstract

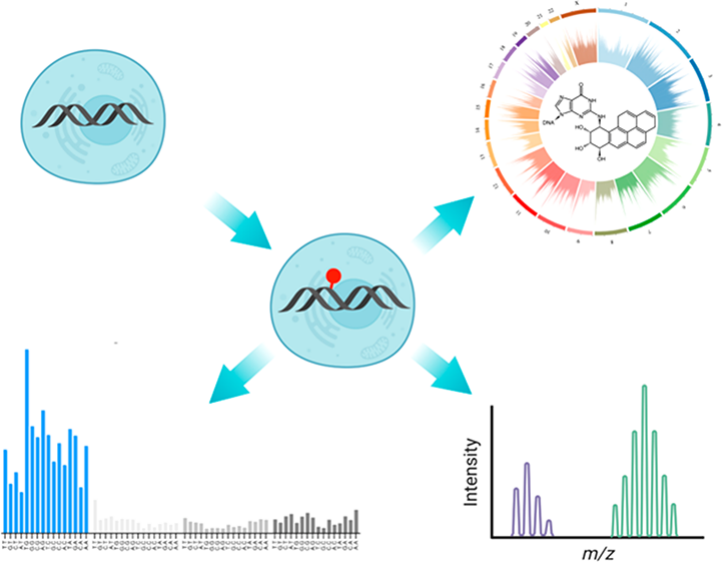

Chemical modifications to DNA bases, including DNA adducts arising from reactions with electrophilic chemicals, are well-known to impact cell growth, miscode during replication, and influence disease etiology. However, knowledge of how genomic sequences and structures influence the accumulation of alkylated DNA bases is not broadly characterized with high resolution, nor have these patterns been linked with overall quantities of modified bases in the genome. For benzo(a) pyrene (BaP), a ubiquitous environmental carcinogen, we developed a single-nucleotide resolution damage sequencing method to map in a human lung cell line the main mutagenic adduct arising from BaP. Furthermore, we combined this analysis with quantitative mass spectrometry to evaluate the dose–response profile of adduct formation. By comparing damage abundance with DNase hypersensitive sites, transcription levels, and other genome annotation data, we found that although overall adduct levels rose with increasing chemical exposure concentration, genomic distribution patterns consistently correlated with chromatin state and transcriptional status. Moreover, due to the single nucleotide resolution characteristics of this DNA damage map, we could determine preferred DNA triad sequence contexts for alkylation accumulation, revealing a characteristic DNA damage signature. This new BaP damage signature had a profile highly similar to mutational signatures identified previously in lung cancer genomes from smokers. Thus, these data provide insight on how genomic features shape the accumulation of alkylation products in the genome and predictive strategies for linking single-nucleotide resolution in vitro damage maps with human cancer mutations.

## Abstract 2

A genome-wide single nucleotide resolution map of the reaction of a tobacco carcinogen with DNA, plus damage quantification, pinpoints where DNA modifications accumulate en route to mutational signatures in human cancers.

Genomic integrity is constantly challenged by chemical alterations to DNA bases, which can influence the growth and function of cells. DNA adducts arise from spontaneous reactions of electrophilic chemicals with nucleobases. They are often miscoding during replication, and thus, their accumulation in the genome is a basis for mutagenesis.^1,2^ Although there is a well-characterized model of causality between DNA adduct formation, evasion of repair, error-prone replicative bypass, and accumulation of genomic mutations,^3,4^ identifying the genomic locations of specific chemically damaged nucleotides has been traditionally impossible, due to their extremely low abundance in the human genome. Therefore, assessment of chemical genotoxicity potential has relied on quantitation of overall increase in DNA damage levels, such as determined by mass spectrometry following the hydrolysis of DNA samples.^5^ However, it is recently possible to map some key base modifications, such as 8-oxoguanine (8-oxoG),^6,7^ abasic sites,^8^ cisplatin cross-links^9^ and UV photodimers,^10,11^ as well as ribonucleotides,^12^ single-, and double-strand breaks,^13,14^ using custom library preparation protocols followed by next-generation sequencing technologies.^15^

Different environmental chemical exposures and drugs induce biological effects by alkylation of DNA. Among these, benzo[a]pyrene (BaP), found in food, coal tar, and cigarette and industrial smoke, is as a known human carcinogen (IARC Group 1A).^16,17^ BaP is metabolized by cytochrome enzymes CYP1A1 and CYP1B1, giving rise to four stereoisomers of the highly reactive BaP-diol-epoxide (BPDE).^18,19^ These stereoisomers can irreversibly react with guanosine in a trans- or cis-ring-opening manner leading to 16 distinct BPDE-DNA adducts, with *N*^2^-trans-(+)-anti-BPDE-deoxyguanosine (*N*^2^-BPDE-dG) being the most abundant and most mutagenic (Figure 1a).^20−22^ BPDE-DNA adducts are removed by nucleotide excision repair (NER),^23^ but those evading repair and bypassed by translesion synthesis polymerases often lead to CG > AT mutations,^24^ which are predominant in cancer driver genes from lung cancers of smokers.^25−27^ A mutational signature arises in cells exposed in vitro to BaP, and it is highly similar to mutational signature 4 found in lung cancers in smokers.^27,28^ However, the relationship between the genomic distribution of DNA adducts with mutational signatures in human cancers has not been addressed for any chemical alkylation-derived adducts, including BPDE-DNA.

**Figure 1:**
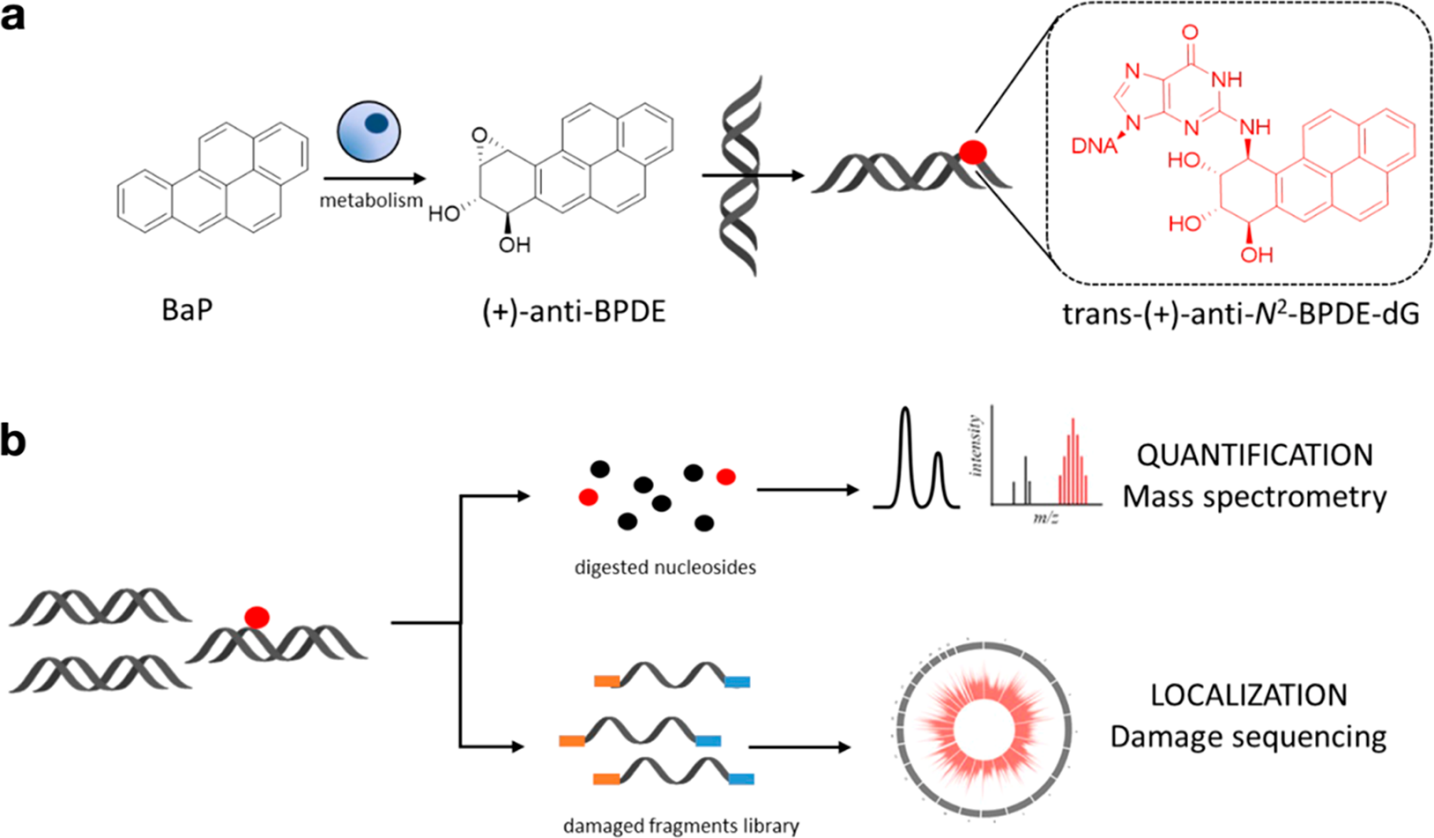
(a) Benzo[a]pyrene (BaP) metabolism yields four stereoisomers of benzo[a]pyrene-diol-epoxide (BPDE): (+)-anti (shown), (−)-anti, (+)-syn, and (−)-syn. The metabolite reacts with DNA and undergoes trans- (shown) or cis-ring opening, leading to the formation of 8 potential isomers. The trans-(+)-anti-N^2^-BPDE-dG adduct shown is the most abundant and mutagenic. (b) Top: *N*^2^-BPDE-dG quantification workflow, involving enzymatic hydrolysis of genomic DNA, followed by chromatographic separation and quantification of deoxynucleosides by LC-MS/MS. In this study, cells were exposed to (±)-anti-BPDE, and total *N*^2^-BPDE-dG was quantified. Bottom: N^2^-BPDE-dG-sequencing workflow, involving denaturation and immunoprecipitation of genomic DNA, followed by marking of adduct sites by DNA extension synthesis with Q5 DNA polymerase. Amplified fragments are sequenced, and the DNA adducts are located at the −1 position relative to read starts.

Evaluating DNA damage profiles with mutational signatures requires the availability of single nucleotide resolution DNA damage maps, and while none exist for *N*^2^-BPDE-dG, its distribution has been evaluated by indirect means via the characterization of genome-wide NER maps gained by development and application tXR-seq.^29^ This method involves immunoprecipitation of the NER core complex to enrich excised DNA fragments containing NER-labile adducts, followed by amplification of these fragments. When human lymphocytes were exposed to BPDE for 1 h, it appeared that *N*^2^-BPDE-dG occurred frequently at CpG sites,^29^ and that in these cells, *N*^2^-BPDE-dG repair was slower than the repair of UV-induced pyrimidine (6–4) pyrimidone photoproducts, but faster than cyclobutane pyrimidine dimers (CPD). Since the excised DNA fragments mapped in tXR-seq vary in length, single nucleotide resolution damage signatures cannot be extracted. Nonetheless, high fidelity DNA polymerase stalling has been shown to be effective for single nucleotide resolution marking of UV-induced cross-links,^29^ but such an approach has not been tested on DNA alkylation adducts such as *N*^2^-BPDE-dG.

In this study, we defined a first single-nucleotide resolution genome-wide map of *N*^2^-BPDE-dG in human lung cells and elucidated relationships between DNA adducts and local sequence contexts, genomic features, and mutational signatures associated with smoking-related lung cancers, as well as how these relationships may be modulated by increasing chemical exposure concentrations. An *N*^2^-BPDE-dG antibody was used to enrich alkylated DNA fragments, and then a high-fidelity polymerase was used to replicate DNA, stalling at and marking the locations of *N*^2^-BPDE-dG adducts. We compared the damage distribution in genomes from a BPDE-exposed human lung epithelial cell line (BEAS-2B) with chemically exposed naked DNA, and additionally used mass spectrometry to quantify corresponding global levels of *N*^2^-BPDE-dG and their dose–response relationship in cells.^30^ Furthermore, we assessed the chromatin state-dependent localization of *N*^2^-BPDE-dG and the strand-bias in its distribution. Finally, we resolved the preferred local sequence contexts for *N*^2^-BPDE-dG as a basis of a DNA damage signature for BaP and compared this profile with mutational signatures extracted from smoking-associated human lung cancers. These findings suggest single-nucleotide-resolution damage sequencing may be used to identify factors promoting accumulation of chemically induced modifications in the human genome.

## Results

### Quantification and Sequencing of *N*^*2*^-BPDE-dG in Human Bronchial Epithelial Cells

**Figure 2:**
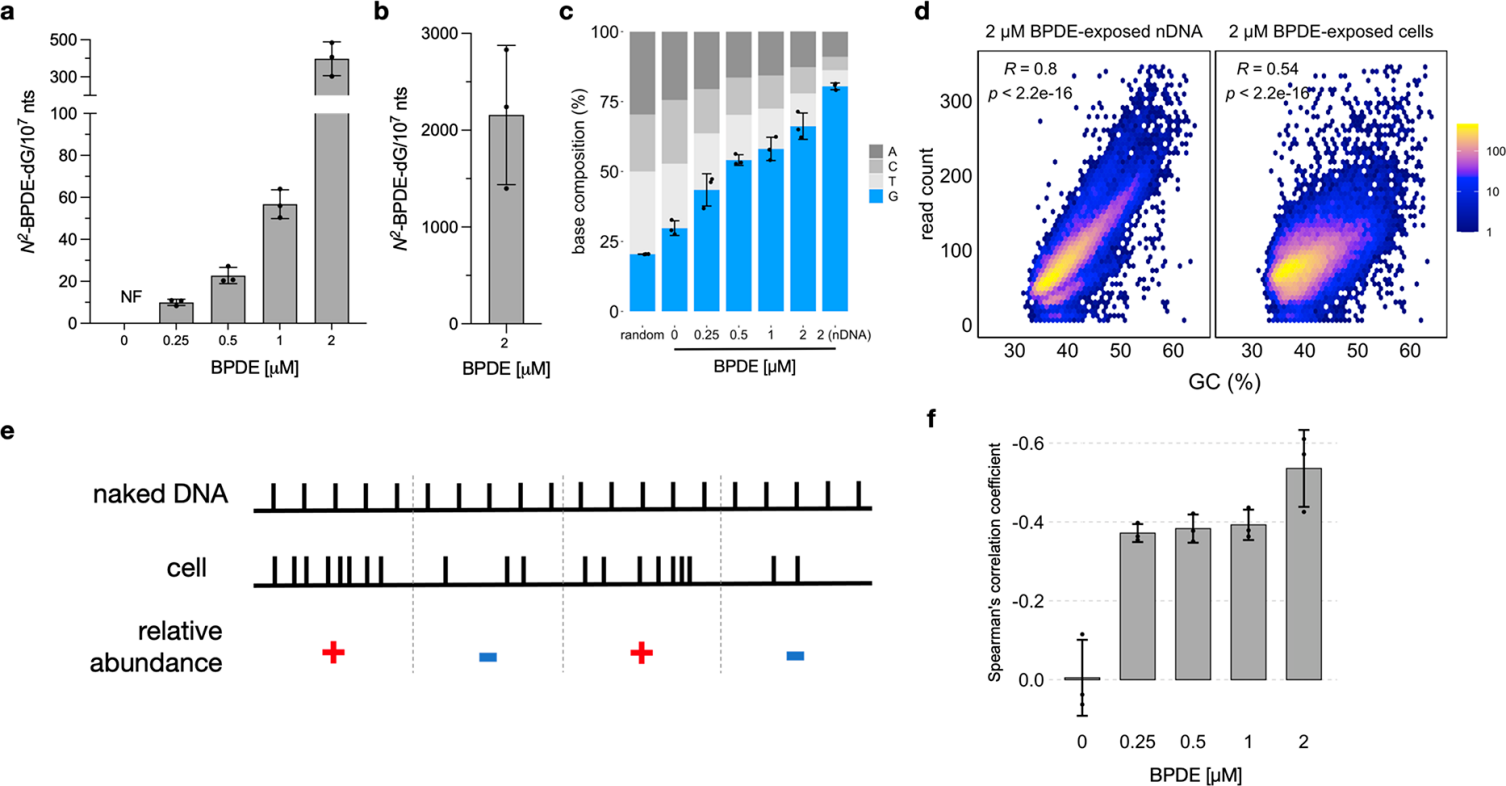
*N*^2^-BPDE-dG quantification and sequencing. Results shown are calculated across three biological replicates ± SD (a) *N*^2^-BPDE-dG levels in BEAS-2B cells exposed to increasing concentrations of BPDE. (b) *N*^2^-BPDE-dG in naked DNA (nDNA) reacted with 2 μM BPDE. (c) Base composition at damage sites. Random values were calculated from three simulated data sets; each contains 10 million random reads across the human genome. (d) Scatter plot showing sequencing read distribution and its Spearman’s correlation coefficient with GC content for nDNA reacted with 2 μM BPDE or cells exposed to 2 μM BPDE. Results were calculated in 100 kb bins across the genome, averaged across three biological replicates. (e) Conceptual visualization of the relative abundance variable evaluated in this study. (f) Bar plots showing the correlation between relative abundance of *N*^2^-BPDE-dG and GC-content.

To determine the dose–response relationship between chemical exposure and *N*^2^-BPDE-dG levels and to relate it with its genomic distribution and sequence context preferences across the entire genome, we used BPDE, the CYP metabolite of BaP, to induce *N*^2^-BPDE-dG in BEAS-2B human bronchial epithelial cells. The use of BPDE (specifically (±)-anti-BPDE) instead of BaP, effectively avoided the aggregation of chemicals in cell culture medium due to the low water solubility of BaP, ensured a uniform exposure, and excluded the influence of cellular metabolism on the exposure chemical. We quantified *N*^2^-BPDE-dG levels by liquid chromatography tandem mass spectrometry (LC-MS/MS) and characterized its distribution by adaptation of HS-Damage-seq^28^ (Figure 1b). To calibrate the working exposure concentrations, we first assessed the sensitivity of BEAS-2B cells toward BPDE by measuring the intracellular ATP content after a 24 h exposure to various concentrations of BPDE (Figure S1). Cell viability remained over 80% for BPDE exposures up to 2 μM. Hence, we quantified the *N*^2^-BPDE-dG resulting from exposure to increasing amounts of BPDE up to that concentration (0.25–2 μM). Genomic DNA was extracted, enzymatically hydrolyzed, and extracted with 1-butanol, and the resulting samples were analyzed by LC-MS/MS. The main DNA adduct is trans-(+)-anti-*N*^2^-BPDE-dG; nevertheless, there are eight isomers that can form when cells are treated with BaP, and using (±)-anti-BPDE as reactant, lower amounts of the other three isomers form (Figure S2). These were quantified as total *N*^2^-BPDE-dG relative to a ^13^C-labeled internal standard mix of the four isomors resulting from the reaction of dG with (±)-anti-BPDE (Figure S2). Thus, in BPDE-exposed samples, we observed a concentration-dependent increase in total *N*^2^-BPDE-dG with values reaching 400 *N*^2^-BPDE-dG/10^7^ nucleotides for 2 μM BPDE (Figure 2A). Additionally, we exposed naked DNA extracted from BEAS-2B (nDNA) cells to 2 μM BPDE as a reference for damage distribution without the influence of DNA packaging or DNA repair. The BPDE-exposed nDNA had 2,000 *N*^2^-BPDE-dG/10^7^ nucleotides (Figure 2B).

Having quantified the global genomic levels of *N*^2^-BPDE-dG upon exposure to BPDE, we examined the antibody’s binding capacity to *N*^2^-BPDE-dG (Figure S3A), optimized the immunoprecipitation conditions specific to this antibody, and refined the library preparation protocol to streamline the process and improve the final yield (see BPDE-dG-Damage-seq library preparation in the Materials and methods section in the Supporting Information). We also characterized the stalling behavior of Q5 polymerase at *N*^2^-BPDE-dG using a synthetically modified oligonucleotide (Figure S3B). The main extension product resulted from stalling of the polymerase immediately before BPDE-dG; however, there was also a minor band corresponding to a premature stall two bases before the adduct. Next, we combined the antibody pull-down with polymerase stalling to prepare libraries from the identical samples used for *N*^*2*^-BPDE-dG quantification. After sequencing, we extracted the genomic coordinates of the damage and examined its enrichment level (Figure 2c). To determine the natural composition of bases, we simulated random reads from the genomes and found a frequency of 20% G, exactly as expected.^31^ In negative control samples, i.e., from nonexposed cells, the four bases were enriched to a similar extent, but with slightly increased frequency of G (30%), suggesting Q5 polymerase may stall more frequently at G, possibly because G is a main target of endogenous DNA modifications,^32^ which could also stall the polymerase. In BPDE-exposed samples, similar to the results of LC-MS/MS quantification, a concentration-dependent increase of G up to 65% at the position called as an adduct was observed in genomic DNA from exposed cells, while BPDE-modified nDNA exhibited the highest frequency of G at the called damage site, reaching 80% (Figure 2c). Considering the propensity for the polymerase to stall one base before the adduct observed using an oligonucleotide template, we probed the data for evidence of the elevation of G frequency at the site where damage would appear in the case of premature stalling, i.e., the −2 position relative to the 5′ end of the read. We found that in comparison to the profound increase in the fraction of G fraction at the −1 position, the frequency of G at the −2 position was almost unresponsive to elevating BPDE levels (Figure S3C–D), which suggests minor incidence of polymerase premature stalling. In addition, the general lack of nucleotide enrichment at sites adjacent to the damaged one (Figure S3E) suggested no preference for the antibody to be biased by DNA sequence context surrounding the damage.

### The Formation and Repair of *N*^*2*^-BPDE-dG Depends on GC Content

To explore the relationship between damage distribution and genomic features, various bin sizes were tested for data analysis (5–100 kb). Owing to the inherently low coverage of damage-sequencing data, data binning is key, as too small bins result in many empty bins, which mask signal distribution patterns, while too large bins average out any signal. By applying the model developed by Gusnanto et al.,^33^ we found low Akaike information criterion in 20–100 kb bins, which means the data analysis results retain the most information at these bin sizes (Figure S4). We also tested smaller bin sizes and found that significant differences between exposed and nonexposed cells were maintained down to 5 kb. Therefore, 5–100 kb bins were used for all analyses; 5 and 100 kb data are shown. A similar bin size also was used effectively for apurinic site sequencing, previously reported by Poetsch et al.^8^ Thereafter, we examined the distribution of *N*^2^-BPDE-dG in naked DNA, and, as expected, we observed a strong GC-dependent distribution of *N*^2^-BPDE-dG along the genome in exposed nDNA (*R* = 0.8, Figure 2d), indicating that GC content is the major driving factor for *N*^2^-BPDE-dG formation in the absence of chromatin structure or repair factors.

We next evaluated the genomic distribution of *N*^2^-BPDE-dG by comparing the enriched regions from chemically exposed nDNA to those in chemically exposed cells, where factors such as chromatin structure and DNA repair mechanisms may eventually shape *N*^2^-BPDE-dG distribution. Thus, we calculated the log_2_-fold change between average damage levels from exposed cells vs exposed nDNA in 100 kb bins [log_2_ (Cell/nDNA)] as the relative abundance of *N*^2^-BPDE-dG, wherein bins with positive values indicate accumulation of *N*^2^-BPDE-dG in cellular genomic DNA, and bins with negative values indicate their depletion (Figure 2e), compared to nDNA. For all chemically exposed cells, there was a negative correlation between this relative abundance value and GC content (Figure 2f, Figure S5A–E). The exception was untreated cells, where no correlation was observed. Additionally, we calculated the correlation coefficients using varying bin sizes from 100 to 5 kb and observed consistent negative coefficients for BPDE-exposed cells, and a significant lower correlation coefficient for the negative control (Figure S5F). These observations suggest that although *N*^2^-BPDE-dG is more easily formed in regions with high GC content, it also appears to be more easily depleted in the same high GC content regions in cells.

### *N*^2^-BPDE-dG Is Reduced in Open Genomic Regions and Highly Expressed Genes

**Figure 3:**
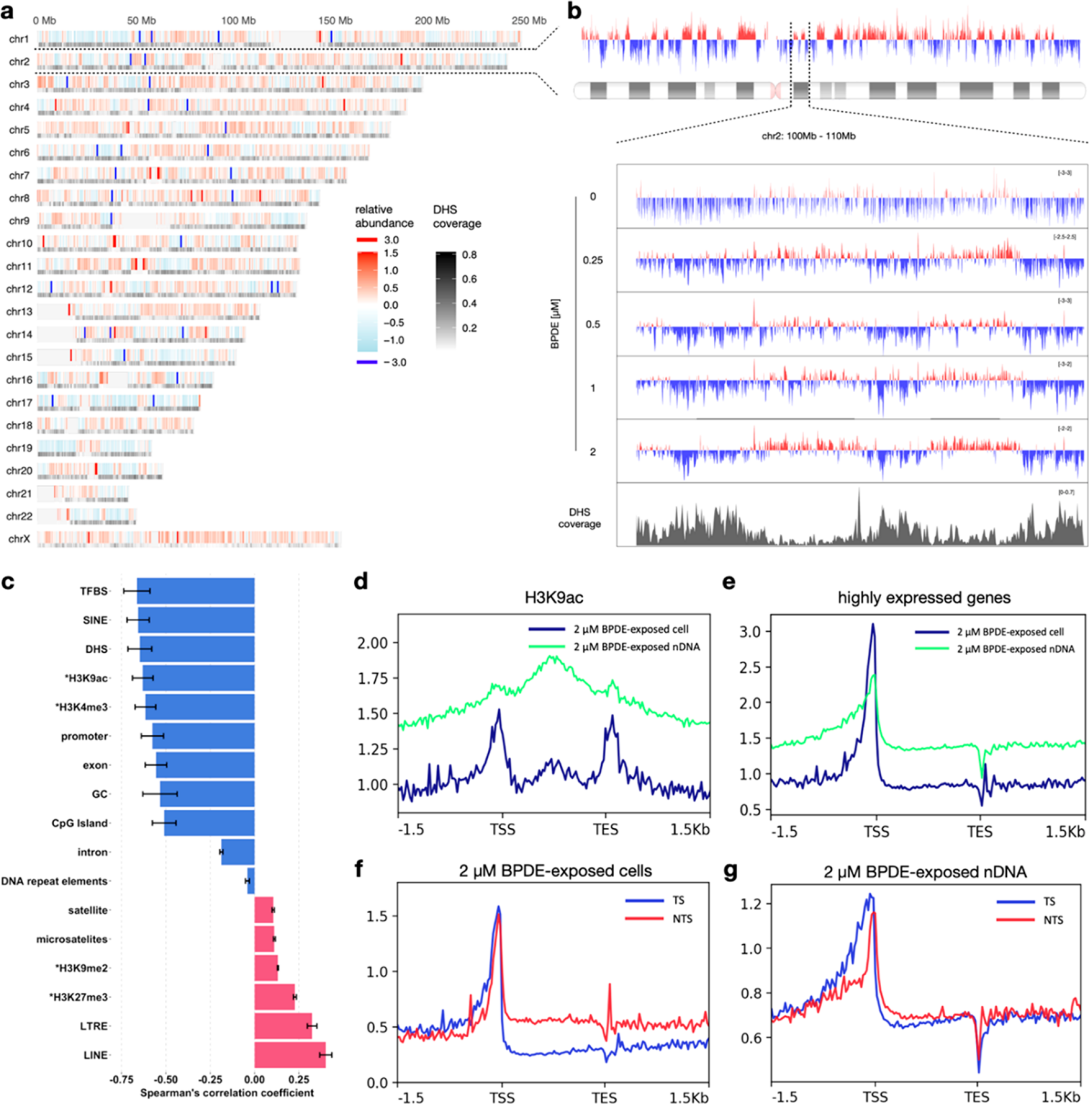
Distribution of *N*^2^-BPDE-dG in genomic DNA. (a) Genome-wide map of *N*^2^-BPDE-dG in cells exposed to 2 μM BPDE. Relative abundance of *N*^2^-BPDE-dG is shown as a function of genomic location. Color scale represents the mean of relative abundance (log_2_ (Cell/nDNA)) of *N*^2^-BPDE-dG calculated in 100 kb bins across three biological replicates. Grayscale represents the DHS coverage in 100 kb bins across genome. Undefined sequences in the human genome annotated in the ENCODE Blacklist,^35^ such as centromeres and telomeres, were removed from the data and are shown in light gray. (b) Detailed profile of the relative abundance of *N*^2^-BPDE-dG on chromosome 2; the view is further expanded at 100–110 Mb for each exposure condition. *Y*-axis represents relative abundance of *N*^2^-BPDE-dG. Data shown are the average of three biological replicates (calculations performed with 5 kb bins across chromosome 2). The plots were smoothed using LOESS (locally estimated scatterplot smoothing). (c) Spearman’s correlation coefficients of relative abundance of *N*^2^-BPDE-dG and genomic features in cells exposed to 2 μM BPDE. Calculations were performed with 100 kb bins across the genome, averaged across three biological replicates ± SD. *ChIP-seq data for BEAS-2B cells were previously published.^29,34,36−38^ (d, e) Profiles of *N*^2^-BPDE-dG in H3K9ac and highly expressed gene regions (*n* = 10,612) from cells and nDNA reacted with 2 μM BPDE. Calculations were performed in 25 bp bins and averaged across three biological replicates. (f, g) Profiles of *N*^2^-BPDE on the transcribed strand (TS) or nontranscribed strand (NTS) of highly expressed genes (*n* = 10,612). Shown are data for cells (f) and nDNA (g) exposed to 2 μM BPDE. Highly expressed genes were defined using mRNA-seq data acquired in this study (see RNA-sequencing and data processing in Methods section in Supporting Information). Data represent mean values calculated from three replicates in 25 bp bin size.

Having established that the overall genomic distribution patterns were robust across chemically exposed cells, we aimed to examine whether damage was accumulated or depleted in a range of different genomic features. As an example, we correlated the distribution of the relative abundance of damage with the accessibility of the genomic regions. Owing to the lack of chromatin accessibility data for BEAS-2B cells, we used the clustered DNase hypersensitive site (DHS) map, identified from 125 different human cell and tissue types, which represents the general accessible chromatin landscape of the human genome.^34^ We then correlated the relative abundance of *N*^2^-BPDE-dG from 2 μM BPDE-exposed cells with the coverage of DHS in 100 kb bins (Figure 3a). There was a negative correlation coefficient (*R* = −0.67 ± 0.06) between the two signals, indicating depletion of damage in open chromatin regions. Similar correlations were also found in cells exposed to lower BPDE concentrations, but not negative controls (Figure S6A–F). The genome-wide map of *N*^2^-BPDE-dG also reveals damage hot spots (relative abundance >99.99 percentile) and cold spots (relative abundance <0.01 percentile). Hot spots occurred more frequently around centromere regions, for instance, in chromosomes 7, 10, 11, and 12. However, no specific pattern was found in the distribution of cold spots. Additionally, chromosomes 17 and 19 contained the most bins with negative relative abundance (>76%), suggesting these two chromosomes are potentially less damaged during exposure or more efficiently repaired afterward. On the contrary, chromosomes 13 and X contained the least bins with negative relative abundance (<29%). Next, we examined the correlation between relative abundance and DHS coverage using bin sizes down to 5 kb. Consistent with previous results, negative correlations were found in all exposed samples, but the correlation was lower due to the smaller bin size (Figure S6G). As an example, we provided the detailed distribution of relative abundance on chromosome 2 and further zoomed into a 10 Mb region for all experimental conditions (Figure 3b), where the correlation between relative abundance and DHS coverage remained discernible.

As DNA methylation levels correlate with genome accessibility, we also carried out bisulfite sequencing and related the damage levels to methylation status of DNA regions. Low methylation regions (LMR) of the genome, including non- and partly methylated regions, accounted for about 12% of the total genome, and fully methylated regions (FMR) accounted for about 88%. LMR and FMR were essentially the same as in the unexposed cells (90% overlap of LMRs, 99% overlap of FMRs), suggesting the BPDE exposure, at least after 24 h, may not alter genome accessibility significantly. Moreover, in LMRs, corresponding to active genomic regions like promoters, there was less DNA damage observed in BPDE-exposed cells than in naked DNA exposed to BPDE (Figure S11). On the other hand, in FMRs, which are associated with gene bodies and repetitive elements, the damage levels were similar (Figure S11).

In addition to DHS and methylation status, other genomic features may also influence *N*^2^-BPDE-dG distribution. Therefore, we analyzed in total 17 genomic features including transcription-associated genomic regions, histone marks, and DNA repeats to better understand the damage distribution pattern along the genome (Figure 3c). Based on the data from 2 μM BPDE-exposed cells, the relative abundance showed negative correlation coefficients with transcription-associated regions, including transcription factor binding sites (TFBS, *R* = −0.66 ± 0.07), promoters (*R* = −0.58 ± 0.06), exons (*R* = −0.56 ± 0.06), CpG islands (*R* = −0.51 ± 0.07), and active histone marks H3K9ac (*R* = −0.63 ± 0.06) and H3K4me3 (*R* = −0.61 ± 0.06), suggesting the depletion of *N*^2^-BPDE-dG in transcriptionally active regions and active regulatory elements. In contrast, the correlation coefficients were positive with the polycomb group associated mark H3K27me3 (*R* = 0.2 ± 0.008) and the heterochromatin mark H3K9me2 (*R* = 0.13 ± 0.003), implying a higher amount of *N*^2^-BPDE-dG in less active genomic regions. As corroborations, we produced detailed damage profiles in different genomic regions from 2 μM BPDE-exposed cells and exposed nDNA, which showed the differences of *N*^2^-BPDE-dG levels (Figure 3d, Figure S7A,B). It is worth noting that there were diametrically opposed relative abundance profiles in the two major classifications of nonlong terminal repeat retrotransposons, including short interspersed nuclear elements (SINEs, *R* = −0.66 ± 0.06) and long interspersed nuclear elements (LINEs, *R* = 0.4 ± 0.03) (Figure 3c). Considering the generally negative correlations between *N*^2^-BPDE-dG relative abundance and the transcriptionally active regions, SINEs are potentially involved more in the genomic activities in the studied cell model, while LINEs are the opposite. In a correlation heat map overview of the association between *N*^2^-BPDE-dG levels, *N*^2^-BPDE-dG relative abundance, and genomic features (Figure S8A), transcription-related features clustered, suggesting that the genomic landscape of *N*^2^-BPDE-dG was not shaped by any single factor, but rather by the combined influence of multiple genomic features.

To analyze the influence of gene expression levels on DNA damage distribution, we used mRNA-seq to obtain the genomic coordinates of common high (*n* = 10,612) or low (*n* = 22,645) expression genes (Figure S7C). While damage levels were similar in low expression genes from both cell and naked DNA samples (Figure S7D), they were generally lower in highly expressed genes from exposed cells vs nDNA (Figure 3e). Interestingly, damage clearly accumulated in promoter regions of highly expressed genes, and peaked approximately 100 bp upstream of the transcription starting sites. Additionally, we found a significantly lower damage level (*P* < 0.0001) in the transcribed strand (TS) than in the nontranscribed strand (NTS) in highly expressed genes from BPDE-exposed samples (Figure 3f) but not in nDNA (*P* = 0.5025, Figures 3g, S8B), suggesting that a bias in strand-specific DNA regulations like TC-NER.

### *N*^2^-BPDE-dG Trinucleotide Sequence Preferences Correlate with Tobacco Mutational Signatures

**Figure 4:**
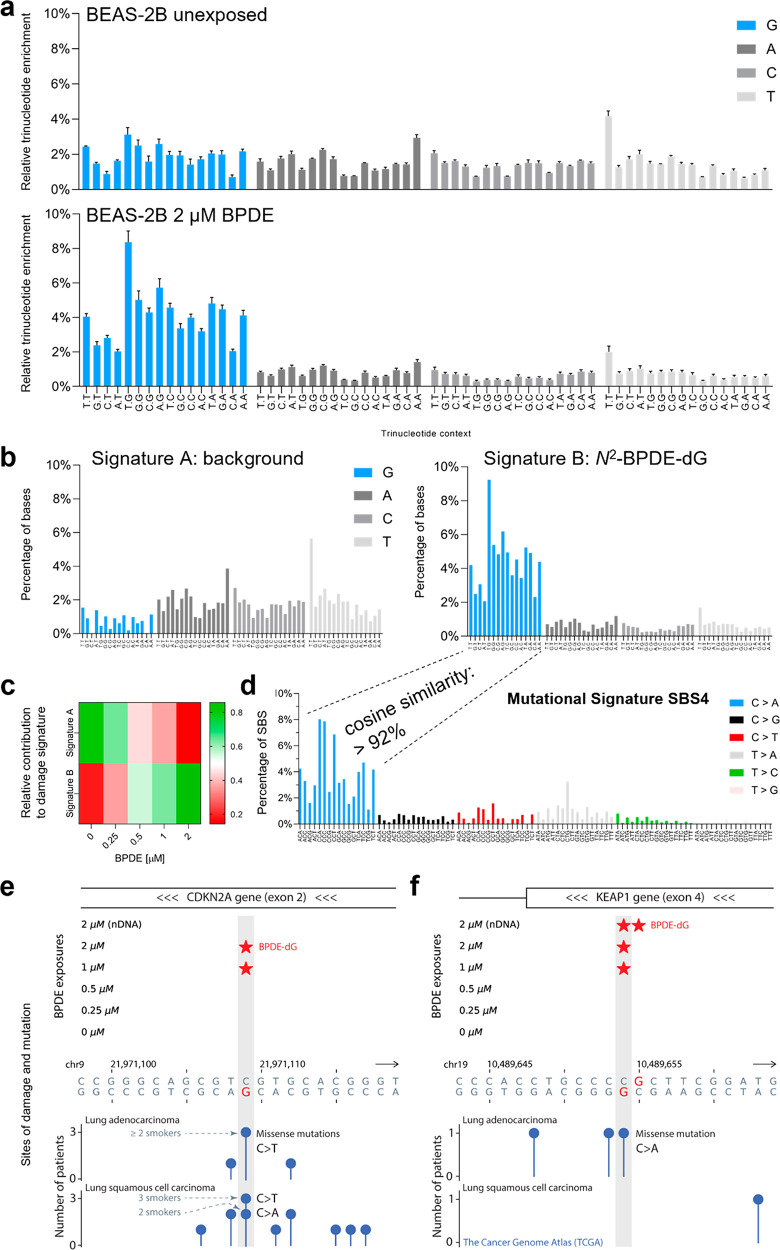
(a) Relative trinucleotide enrichment at the predicted damage site from nonexposed cells and cells exposed to 2 μM BPDE. (b) Signatures extracted from all samples using a non-negative matrix factorization. Two signatures were extracted and labeled A and B. (c) Heat map representing the relative contribution of Signature A and B to the damage signature extracted from cells exposed to different BPDE concentrations. (d) Mutational Signature 4 extracted from smoking-related lung cancers. Cosine similarity of the extracted relative trinucleotide enrichment profile with C > A mutations in Signature 4. (e–f) BPDE-dG at lung-cancer-associated mutation sites located in tumor suppressor genes CDKN2A (e) and KEAP1 (f). A star indicates BPDE damage called by at least two reads across experimental replicates of a given condition. The identified damage-mutation match in CDKN2A is the most frequently mutated site of this gene in lung adenocarcinomas. As a reference for the *Y*-axis values of the mutation data in KEAP1, for ∼93% of lung-carcinoma mutation sites the number of patients with mutation is one, and for the remaining mutation sites this number is two. The TCGA mutational data were obtained via UCSC Table Browser. The gene annotation is according to GENCODE V41 (MANE-only set).

*N*^2^-BPDE-dG damage sequencing revealed preferential damage accumulation in certain genomic regions (Figure 3); due to data binning, however, information on the relevance of local DNA sequence contexts that may influence DNA damage formation, and repair was not yet fully utilized in this aspect of our analysis. Mutations in human cancer genomes have been classified into signatures of 96 bar plots of the six possible base substitutions in trinucleotide contexts.^39^ Signatures of the persistence of DNA damage in trinucleotide contexts (by analogy, DNA damage signatures) may therefore be a potential prognostic marker to identify mutational processes. Thus, we plotted the relative trinucleotide enrichment of the four bases at the damage sites (Figure 4a and Figure S9). We found that the trinucleotide enrichment from nonexposed cells had a high background, as evidenced by the equal occurrence frequency of each of the four bases. In contrast, enrichment of G was found for the trinucleotide context from BPDE-exposed cells, revealing the preferential contexts for *N*^2^-BPDE-dG distribution.

Non-negative matrix factorization was used to extract mutational signatures from millions of human cancer trinucleotide background mutation data, to isolate single mutagenic processes and use them to identify and classify the cause of human cancers. By using the same approach, we extracted a background signature, referred to as “signature A”, represented mainly in nonexposed cells (Figure 4b). The frequencies and the lack of features in signature A suggest that it reflects the background noise resulting from the library preparation. In addition, we also extracted the damage signature B, which contains a distinctive G profile that is highly present in BPDE-exposed cells (Figure 4b). Furthermore, signature B was found to increase in representation with increasing concentration of BPDE (Figure 4c).

We used cosine similarity as a measure to compare the experimentally determined damage signature to mutational signatures extracted from human cancer genomes. Given that *N*^2^-BPDE-dG mainly leads to C > A mutations, we compared the 16 bar corresponding to damaged guanosines in each trinucleotide context, with the frequencies of 16 C > A bars from mutational signature 4.^40^ The results showed that the profile of *N*^2^-BPDE-dG damage was highly similar (>0.92 cosine) with the mutational signature 4 (Figure 4d), which was mainly observed in tobacco smoking-associated lung cancers. Additionally, we also compared the *N*^2^-BPDE-dG profile with all other available mutational signatures from humans and found a relatively lower cosine similarity (coefficient < 0.80, Figure S10).

### *N*^2^-BPDE-dG Was Found at Lung Cancer Mutation Sites in Tumor Suppressor Genes

Having observed a high similarity between the experimental *N*^2^-BPDE-dG damage distribution and a cancer mutational signature, we next set out to explore if some of the damage sites detected in BPDE-exposed cells match, at the single-nucleotide level, specific mutation sites found in lung cancer patients. To test for damage-mutation matches, we focused on mutation sites from The Cancer Genome Atlas (TCGA) in 18 statistically significant mutated genes of lung adenocarcinomas.^41^ From these, *N*^2^-BPDE-dG was detected at least twice, i.e., in at least two chemical exposure conditions, in four genes, namely, the chromatin modifying gene SMARCA4 and the tumor suppressor genes CDKN2A, KEAP1 and TP53 (two damage-mutation matches in TP53 and one in each of the other genes). Increasing the stringency of the analysis, among these, we selected two mutation sites located in tumor suppressor genes CDKN2A (Figure 4e) and KEAP1 (Figure 4f) as they had the BPDE damage called by at least two reads across experimental replicates of a given condition. The identified damage-mutation match in CDKN2A was the most frequently mutated site of this gene in the lung adenocarcinoma data, and one of the top 0.007% (28/381,737) most frequently mutated sites genome-wide in lung carcinomas (TCGA). Almost all lung-carcinoma patients with mutations at this site, where we consistently observed *N*^2^-BPDE-dG in exposed cells, were reported to be smokers (Figure 4e).

## Discussion

Genome-wide distribution profiles of DNA alkylation adducts as potentially predictive markers of carcinogenic hazards have not been widely investigated. Therefore, in this study, we defined the overall abundance of *N*^2^-BPDE-dG in a chemically exposed human cell line and also determined the genome-wide distribution of this adduct at a single-nucleotide resolution, under the same conditions. We evaluated this genome-wide map of *N*^2^-BPDE-dG by assessing the association of predominant damage locations with chromatin accessibility and the DNA strand identity. We observed a concentration-dependent increase of *N*^2^-BPDE-dG in BPDE-exposed human bronchial epithelium cells but a rather stable pattern of *N*^2^-BPDE-dG distribution across the genome at both high and low exposure levels. Furthermore, we identified preferred flanking nucleotide sequence contexts of *N*^2^-BPDE-dG that shared similar features with mutational signatures from human lung cancers, and found evidence for damage formation at specific lung-cancer mutation sites located in tumor suppressor genes.

### BPDE-dG-Damage-seq Is Reproducible, Sensitive, and Specific

In order to characterize the relative abundance of *N*^2^-BPDE-dG in genomic DNA of human lung cells, we compared the damage distribution patterns in BPDE-exposed cells to those in BPDE exposed naked DNA. We found consistent correlations of relative abundance of *N*^2^-BPDE-dG with various genomic features across triplicate experiments. Additionally, the consistency was stable under different analysis window sizes ranging from 5 to 100 kb (Figures S5, S6). These metrics suggested a high reproducibility of the method.

To relate the quantities of damage to its distribution patterns, we used LC/MS-MS analysis of total adduct burden in the genome, thus combining damage quantification and location mapping data in the same biological context, for the first time, to the best of our knowledge. The amount of *N*^2^-BPDE-dG induced by BPDE exposure *in vitro* is a complex interplay of concentration, exposure duration, and cell types and generally ranges between 10 and 500 adducts per 10^7^ nucleotides,^42,43^ which is the range our *N*^2^-BPDE-dG quantification data (10–400 *N*^2^-BPDE-dG/10^7^ nt) fall into. Though previous studies that described the genome-wide profiles of CPD did not quantify the damage, the damage level can still be estimated based on published data. For example, in the study by Hu et al., CPD was induced by UVC irradiation at 10 J/m^2^,^30^ anticipated to form more than 1,000 CPD/10^7^ nucleotides.^44−46^ These levels are higher than the *N*^2^-BPDE-dG levels in this study, by about 1 to 2 orders of magnitude. Despite the low relative levels of *N*^2^-BPDE-dG, we still found a consistent damage distribution pattern in cells exposed to varying concentrations of BPDE (Figures S5, S6). The relative abundance of *N*^2^-BPDE-dG was still found to be significantly different from the control group even at the lowest BPDE concentration (10 *N*^2^-BPDE-dG/10^7^ nt), but the results were similar to those obtained with higher exposure concentrations, indicating the high sensitivity and specificity of *N*^2^-BPDE-dG damage sequencing.

### Genome Accessibility Influences *N*^2^-BPDE-dG Distribution

DNA packaging may influence DNA damage profiles in different ways. On the one hand, it has been shown that nucleosome-packed DNA is protected from damage,^47−49^ suggesting that open genomic regions may be more susceptible to damage. For example, open chromatin regions accumulate oxidative damage shortly after exposure to oxidants.^7,8^ On the other hand, open chromatin regions are actively transcribed and dynamically interact with transcription factors and other coactivator complexes, including DNA repair factors. For example, repair of UV-induced DNA damage was found to preferentially initiate in open chromosomal regions.^29,30,50^ In the present study, there was less damage in open vs closed chromatin regions (Figure 3), suggesting that with the combination of long exposure duration (24 h) and high BPDE reactivity though *N*^2^-BPDE-dG adducts could rapidly form, they are also effectively repaired in the open chromatin regions. Further studies involving BPDE adduct mapping in repair-deficient backgrounds would be needed to test this hypothesis.

Correlations between genome accessibility and alkylation damage (Figure 3) are limited by the lack of available DNase-seq data in BEAS-2B cell lines, and reliance on a generic DNase map of the human genome assembled from multiple cell lines. While the distribution of DNase peaks varies in different cell lines, such that a more specific analysis would require DNase data from BEAS-2B cells, we investigated how chemical exposure of the BEAS-2B cells may influence accessibility as it correlates with DNA methylation levels. The regions of the genome categorized as high vs low methylation status overlapped by more than 90% in all cases, and, moreover, there was a correlation between damage accumulation and high methylation status and vice versa. These observations are consistent with associations observed from comparing DNA damage profiles in exposed BEAS-2B cells made on the basis of average cell chromatin accessibility data here, as well as correlations drawn between cell-specific DHS data in previous studies of CPD damage maps by Hu et al.^30^ and the 8-oxoG damage maps by Wu et al.^6^ Unlike transient reactive UV and oxygen radicals, however, BPDE reacts more slowly, and its preassociation with more accessible structural elements of chromatin appears to drive distribution patterns for its reactivity.^47,51,52^ Thus, although open chromosomal regions may be more prone to damage formation, the damage profiles observed in cells after 24 h appeared to be shaped by repair, suggesting that in addition to focusing on repair-deficient damage mapping experiments, these also should be time-resolved to better understand the evolution of persistent damage signatures.

Another interesting observation in the present study was the significant accumulation of *N*^2^-BPDE-dG at the promoter region of the transcription start sites (TSS) of highly expressed genes, followed by a sharp decrease at the TSS (Figure 3E). The promoter regions of highly expressed genes are among the most open regions in the genome, and had relatively low damage levels. We speculate that the significant accumulation of damage before TSS may be due to the frequent interaction between DNA and the transcription preinitiation complex, and the previous observation that the binding of transcription factors to DNA impairs DNA repair.^53,54^ The transcription preinitiation complex consists of up to 100 proteins essential to start gene transcription,^55^ and a peak in damage accumulation was observed in the region of its binding sites. To probe this relationship further, we reanalyzed previously published data tracking NER-mediated removal of *N*^2^-BPDE-dG in the highly expressed genes in GM12878 cell line using tXR-seq^29^ Indeed, NER function appeared to be reduced approximately 100 bp upstream of the TSS (Figure S11) and, NER valleys were observed in the tXR-seq data in the same locations as *N*^2^-BPDE-dG peaks observed in the present study.

### *N*^2^-BPDE-dG Accumulates on the Nontranscribed Strand

It is well established that preferential removal of *N*^2^-BPDE-dG in the TS by TC-NER protects active genes.^29,56^ In the present study, we extracted 10,612 common genes that were highly expressed in all experimental cells based on mRNA-seq and found that among them, there was less *N*^2^-BPDE-dG level on the TS vs NTS (Figure 3F). In addition, we found that the biased distribution of *N*^2^-BPDE-dG between TS and NTS persists for several kb after transcription end sites (TES). This phenomenon may indicate that the effect of TC-NER is not limited to the transcriptional compartment, which may be evaluated in further damage mapping studies in TC-NER-deficient cells.

### BPDE Damage Patterns Share Features with Cancer Mutational Signatures and Hot-Spots

DNA sequence contexts affect chemical reactivity, repair efficiency, and error frequency in translesion DNA synthesis, suggesting that trinucleotide contexts can influence the likelihood of mutagenesis.^4^ The high resolution of the *N*^2^-BPDE-dG damage sequencing data allowed us to assess the DNA damage distribution probabilities in trinucleotide contexts, thus enabling the comparison with human cancer mutational signatures.^39^ Whereas cancer mutational signatures comprised a non-negative matrix of 96 triad sequences, there are 16 possible contexts for guanosine monoadducts. Thus, we compared the experimentally derived values for the frequency of *N*^2^-BPDE-dG formation in each of the 16 possible triads, which we call a DNA damage signature, with COSMIC mutational signatures for each of the possible single base substitutions. The *N*^2^-BPDE-dG damage signature was most similar (92%) with the C > A component of COSMIC mutational signature 4 (Figure S10), which is associated with smoking-related lung cancers. Furthermore, we explored the *N*^2^-BPDE-dG damage sequencing data at the single-nucleotide level and were able to identify damage sites in BPDE-exposed cells that match specific mutation sites found in lung cancer patients. In particular, we consistently observed *N*^*2*^-BPDE-dG at known mutation sites in tumor suppressor (CDKN2A, KEAP1, and TP53) and chromatin modifying (SMARCA4) genes. Future work involving the characterization of DNA damage patterns and their comparison with COSMIC mutational signatures and mutations at specific loci may form a basis for predicting carcinogenic potential of chemical exposures or to unravel the unknown etiologies of mutational processes in human cancers.

## Conclusion

DNA damage maps resulting from the development and application of damage-specific sequencing techniques are rapidly emerging. This study provides the first single-nucleotide resolution map of damage patterns specific to the human carcinogen BaP in human cells. Moreover, it combines quantitative aspects of DNA damage formation throughout the genome, obtained using mass spectrometric analysis with damage-sequencing-derived distribution profiles. While DNA damage signature analysis has potential for predicting mutational signatures, the data herein describe the response of a single cell type, at a fixed time point, providing a snapshot of damage distribution during the dynamic process of DNA damage and repair. Therefore, further work is needed using approaches established here to unravel time-dependent evolution of DNA damage profiles and signatures in cells with diverse genetic and epigenetic characteristics, thus forming a basis for elucidation of genome-wide mutagenesis mechanisms.

## Data Availability

Processed sequencing data have been deposited in the NCBI Gene Expression Omnibus (GEO) (https://www.ncbi.nlm.nih.gov/geo/) under accession no. GSE224001. The code used for data analysis is available at https://gitlab.ethz.ch/yanjiang/bpde.

## Supporting Information Available

Materials and Methods, Figures S1–S13 and Tables S1–S2. Figure S1, Cell viability assay; Figure S2, LC-MS/MS chromatograms; Figure S3, Antibody binding and polymerase stalling capacity; Figure S4, Window size selection; Figure S5, Analysis of GC content influence; Figure S6, Analysis of DHS coverage influence; Figure S7, Damage profiles relative to histone modification marks and gene expression; Figure S8, Relating the damage to genomic features; Figure S9, Trinucleotide damage signatures; Figure S10, Cosine similarity between damage and mutational signatures; Figure S11, Relating the damage to DNA methylation; Figure S12, Comparing the damage distribution to tXR-seq data; Figure S13, Schematic of BPDE-dG damage sequencing; Table S1, Transitions and collision energies used to quantify *N*^*2*^-BPDE-dG by LC-MS/MS; Table S2, Sequences of oligonucleotides (PDF)
