# Correction to “Quantification and Mapping of Alkylation in the Human Genome Reveal Single Nucleotide Resolution Precursors of Mutational Signatures”

**DOI:** 10.1021/acscentsci.3c01597

**Published:** 2024-01-25

**Authors:** Yang Jiang, Cécile Mingard, Sabrina M. Huber, Vakil Takhaveev, Maureen McKeague, Seiichiro Kizaki, Mirjam Schneider, Nathalie Ziegler, Vera Hürlimann, Julia Hoeng, Nicolas Sierro, Nikolai V. Ivanov, Shana J. Sturla

The funding statement is amended to clarify the independent nature of projects from which results are reported in the article:

