# Supplementary material for: Quantification and Mapping of Alkylation in the Human Genome Reveal Single Nucleotide Resolution Precursors of Mutational Signatures
Source: ACS Cent Sci. 2023 Feb 22;9(3):362–72. doi: 10.1021/acscentsci.2c01100 (PMC10037492; doi:10.1021/acscentsci.2c01100)
Supplement: Supplementary file 1 — oc2c01100_si_001.pdf [file oc2c01100_si_001.pdf]

Supporting Information for

Quantification and mapping of alkylation in the human genome reveal single nucleotide resolution precursors of mutational signatures

Yang Jiang*[,1], Cécile Mingard*[,1], Sabrina M. Huber[1], Vakil Takhaveev[1], Maureen McKeague[1,2], Seiichiro Kizaki[1], Mirjam Schneider[1], Nathalie Ziegler[1], Vera Hürlimann[1], Julia Hoeng[3], Nicolas Sierro[3], Nikolai V. Ivanov[3], Shana J. Sturla[1]

*Authors contributed equally to the work

[1]ETH Zurich, Department of Health Sciences and Technology, Schmelzbergstrasse 9, Zurich, CH 8092

[2]McGill University, Pharmacology and Therapeutics; Chemistry, 801 Sherbrooke Street West, Montreal, QC, CAN H3A 0B8

[3]Philip Morris Products SA, Quai Jeanrenaud 3, Neuchatel, CH 2000

Corresponding author: Shana J. Sturla

Address: Schmelzbergstrasse 9, 8092 Zürich, Switzerland

Phone number: +41 44 632 91 75

Email: sturlas@ethz.ch

This PDF file includes:

- Materials and Methods, *pages S2-11*
- Figures S1-S13, *pages S12-22*
- Tables S1-S2, *page S23*

Materials and Methods

Oligonucleotides and indexing primers were purchased from Eurogentec (Seraing, Belgium). BEAS-2B human bronchial epithelial cells were purchased from ATCC (CRL-9609, Manassas, VA, United States) and regularly tested for mycoplasma contamination. Genomic DNA was extracted using the QIAamp DNA Mini kit (Qiagen, Hilden, Germany) according to the manufacturer's instructions. In cases where large amounts of DNA were required, such as naked DNA (nDNA) exposure to BPDE, a DNA isolation kit for cells and tissues (Roche, Basel, Switzerland) was used. DNA concentrations were measured using the Quantus Fluorometer with QuantiFluor ONE dsDNA Dye. N^2-BPDE-[$^{13}C_{10}$]-dG was synthesized as previously reported and provided by Prof. Robert Turesky (University of Minnesota, Minneapolis, USA) (1); the procedure was based on previous reports,(2-4) and the concentration was determined by UV based on the reported extinction coefficient.(3) A BPDE-modified oligonucleotide (Table S2) was prepared as previously reported (5, 6) from the reaction of (±)-anti-BPDE with the corresponding oligonucleotide containing a single G and purification of the main product trans-(+)-anti-N^2-BPDE-dG; it was provided by Prof. Nicholas Geacintov (New York University, New York, USA). (±)-anti-BPDE was from MRIGlobal (Kansas City, USA, MRI #0477); it is an extremely unstable compound used as an anhydrous, inhibitor-free tetrahydrofuran solution, following detailed precautions provided by the manufacturer. It is also reasonably considered to be a carcinogen and should be handled with corresponding precautions.

BEAS-2B* cell *culture and chemical exposure

BEAS-2B cells were cultured in BEGM Bronchial Epithelial Cell Growth Medium Bullet Kit (Lonza, Basel, Switzerland) at 37 °C in a humidified atmosphere with 5% CO_2. BEAS-2B cells grown in 10 cm dishes were exposed to (±)-anti-BPDE (0.25–2 μM) for 24 h. Negative control cells were exposed to 0.1% solvent (anhydrous tetrahydrofuran/5% triethylamine). After exposure, cell pellets from each biological replicate were harvested for DNA or RNA extraction.

Exposure of nDNA to BPDE

Purified genomic DNA from BEAS-2B cells (250 ng/μL, 100 μL) in Tris-HCl buffer (10 mM, pH 8.0) was mixed with 2 μL anhydrous tetrahydrofuran/5% triethylamine solution of (±)-anti-BPDE (100 μM). The mixture was incubated overnight on ThermoMixer (Eppendorf, Hamburg, Germany) at 37

°C, 800 rpm, and purified by ethanol precipitation. A total of 10 µg of purified DNA was used for N^2-BPDE-dG quantification using LC-MS/MS, 1.5 µg was used for damage sequencing (described in N^2-BPDE-dG-Damage-seq library preparation).

Total RNA extraction

Total RNA was extracted from BEAS-2B cells using TRIzol RNA Isolation Reagents (Thermo Fisher Scientific, Waltham, MA, United States). 1.3–1.8 × 10^5 cells were lysed for each extraction and homogenized in 1 mL of TRI-reagent. For each lysis, 0.2 mL chloroform per 1 mL TRI-reagent was added to the sample and vortexed for 15 s. The samples were incubated for 3 min, prior to centrifugation at 12,000 x *g* for 15 min at 4 °C. The aqueous phase was transferred to a new 1.5 mL tube and 0.6 mL of isopropanol per 1 mL of TRIzol reagent was added and mixed gently by inverting the tube several times. The samples were incubated at ambient temperature for 10 min, prior to centrifugation at 12,000 x *g* for 10 min at 4 °C and the supernatant was discarded. The RNA pellets were washed with 75% (v/v) ethanol and centrifuged at 7,500 x *g* for 10 min at 4 °C. The supernatant was removed and the RNA was air dried and resuspended in an appropriate amount of RNase-free water. Samples were flash-frozen in liquid nitrogen and stored at -80 °C.

Enzymatic hydrolysis of DNA and butanol enrichment of N^2-BPDE-dG

DNA was hydrolyzed to yield deoxyribonucleosides by a previously reported procedure (7). Briefly, 0.03 U/10 µg DNA of phosphodiesterase I from *Crotalus adamanteus* venom (Merck KGaA, Darmstadt, Germany), 25 U/10 µg DNA of benzonase nuclease (Merck KGaA), and 20 U/10 µg DNA of alkaline phosphatase from bovine intestinal mucosa (Merck KGaA) were mixed in 50 µL of digestion buffer (20 mM Tris-HCl, 100 mM NaCl, 20 mM $MgCl_2$, pH 7.6) and added to 10 µg dry DNA. Each sample was spiked with 1 pmol of N^2-BPDE-[$^{13}C_{10}$]-dG corresponding to 0.5 µL of a 2 µM stock solution. Samples were incubated at 37 °C for 6 h. After incubation, 450 µL of Milli-Q water was added to reach 500 µL and digestion enzymes were removed by filtration using a polyethersulfone 10 kDa MWCO filter (516-02 VWR, Radnor, PA, United States). An aliquot of the resulting solution (50 µL) was reserved for quantification of dG (see dG quantification section). From the remaining solution, modified nucleosides were enriched by butanol extraction, by a procedure adapted from Klaene *et al.* (8). Per 450 µL sample, 150 µL of water-saturated butanol

(prepared by mixing 3 mL butanol with 300 µL Milli-Q water) was added. After thoroughly vortexing, the sample was centrifuged at 3,000 x *g* for 1 min. The upper butanol layer was transferred to a 1.5 mL DNA LoBind tube (Eppendorf). A second extraction was performed on the same sample by repeating the previous steps. The butanol layers were combined in the same LoBind tube, and additional Milli-Q water (300 µL) was added. The mixture was thoroughly vortexed and centrifuged at 3,000 x *g* for 1 min. The upper layer was transferred to a vial for high-performance liquid chromatography (HPLC) and evaporated to dryness using miVac Centrifugal Concentrator (Genevac, Ipswich, United Kingdom). The resulting residue was resuspended in 20 µL 50% MeOH for further mass spectrometric analysis.

Quantification of dG in isolated DNA

The dG content of enzymatically digested DNA samples was determined by HPLC. Nucleosides were separated with an Agilent 1100 or 1200 series HPLC system (Agilent, Santa Clara, CA, United States) equipped with a Phenomenex Kinetex 2.6 µm C18 100 Å column 150 x 2.1 mm (00F-4462-AN, Phenomenex, Torrance, CA, United States). The injection volume was 20 µL and the nucleotides were detected at 254 nm. Compounds were eluted using solvent A (3% acetonitrile in water) and solvent B (acetonitrile). A gradient elution was applied at a flow rate of 200 µL/min: 0%–15% B (0–6.0 min), 15%–80% B (6.0–6.1 min), 80% B (6.1–11.0 min), 80%–0% B (11.0–11.2 min), followed by re-equilibrium for 15 min. Calibration curves were produced from the analysis of dG in water using six calibration points in the range of 0.1–50 µM. The dG concentration from the 20 µL aliquots of the digestion mix (see DNA digestion) was determined from the calibration curve, and it was used to determine the total number of nucleotides (#nt) in each sample. The calculation assumed a GC content of 41% in the human genome according to the following equation in which V_{tot} is the total volume of the digestion mix in L described in Eq. 1:

$$\#nt = \frac{\left(dG\left(\frac{mol}{L}\right) * V_{tot}\,(L) * N_A\right)}{0.21} \quad \text{(Eq. 1)}$$

Quantification of N^2-BPDE-dG in isolated DNA

Stable isotope dilution LC-MS/MS experiments aiming to identify and quantify N^2-BPDE-dG were performed with a Waters nanoAcquity UPLC system (Waters, Milford, MA, United States) coupled to a TSQ Vantage triple quadrupole mass spectrometer (Thermo Fisher Scientific). N^2-BPDE-dG was resolved on an Acquity BEH130 C18 M-class column 1.7 µm 0.3 mm × 150 mm (SKU: 186007566, Waters) maintained at 40 °C, applying a gradient starting at 90% of 0.1% formic acid in water followed by increasing proportions of 0.1% formic acid in acetonitrile up to 40%, at a flow rate of 5 µL/min over 15 min. An additional 15 min were used to wash and re-equilibrate the column with starting conditions. Separation of N^2-BPDE-dG diastereomers was achieved using isocratic elution with 16% acetonitrile for 65 min. The sample injection volume was set to 4 µL.

The MS was operated in positive electrospray ionization mode and N^2-BPDE-dG was analyzed in selected reaction monitoring mode (SRM) as its $[M+H]^+$ species. The general source-dependent parameters were as follows: capillary temperature: 270 °C, spray voltage: 3,000 V, sheath gas pressure: 10 (arbitrary unit), and collision gas pressure: 1.5 mbar. Tuned S-lens values were used, and the scan width and scan time were set to 0.1 m/z and 0.1 s, respectively. The transitions and product ions used for these measurements are listed in Table S1. Calibration lines were prepared in 50% MeOH for all analytes in the range of 0.25–50,000 nM, using seven calibration points spiked with a final concentration 50 nM $^{13}C_{10}$- N^2-BPDE-dG each. Data were processed using Thermo Xcalibur Quan Browser (Ver. 2.1.0.1139) selecting for internal calibration. The total number of N^2-BPDE-dG in each sample was determined from the calculated concentration according to the Eq. 2:

$$\#N^2\text{-BPDE-dG} = N^2\text{-BPDE-dG}\left(\frac{mol}{L}\right) * V_{tot}\,(L) * N_A \qquad \text{(Eq. 2)}$$

where N^2-BPDE-dG (mol/L) = N^2-BPDE-dG concentration determined via LC-MS/MS-detection, V_{tot} (L) = total volume of mass spec sample and N_A = Avogadro number = $6.022*10^{23}$ mol^{-1}.

Q5 polymerase extension assay

A BPDE-modified 24mer oligo at a single G (Table S2) was mixed with an equal amount (25 pmol) of primer in 10 µL MilliQ water. An equal volume of NEBNext Ultra II Q5 Master Mix was added,

and the mixture was incubated with the following conditions in thermocycler: 50 s at 98 °C, 5 min at 37 °C, and hold at 37 °C. The extension products were mixed with 4 µL gel loading dye (B7024S, New England Biolabs) and separated by 20% denaturing PAGE. The gel was visualized with a gel imager (ChemiDoc MP Imaging System, Bio-Rad).

BPDE-dG-Damage-seq library preparation

Oligonucleotides used in library preparation are listed in Table S2 and a schematic of the library preparation is shown in Figure S13. AD1 and AD2 adaptors (40 µM) were prepared by mixing equal volumes (20 µL) of 100 µM AD1T/AD2T and AD1B/AD2B with 10 µL 5x Annealing buffer (50 mM Tris, pH 8.0, 250 mM NaCl, 5 mM EDTA), heating to 98 °C, then allowing to slowly cool to 25 °C.

Genomic DNA (1.5 µg) extracted from BEAS-2B cells was sheared using a Q800 sonicator (Qsonica, Newtown, CT, United State) to produce fragments of average length 400 bp, using the following program: 20% amplitude for 3 min, 2 s on/5 s off. Fragmented DNA was subjected to size-selective purification to remove fragments smaller than 200 bp with 1x volume of AMPure XP DNA purification beads (Beckman Coulter, Brea, CA, United States). Next, the size-selected DNA (1 µg) was used for end preparation and AD1 (40 µM) ligation according to the instructions of NEBNext Ultra II DNA Library Prep Kit for Illumina (New England Biolabs, Ipswich, MA, United States). The ligation mixture was kept 16 h at 4 °C for adequate ligation. The ligation product was purified with 0.7x volume (65 µL) of AMPure XP and eluted with 12 µL 0.1x TE buffer. Eluted DNA was denatured by mixing with 5 µL of 8 M urea, heated at 98 °C for 2 min, and immediately placed on ice. The denatured DNA was mixed with 0.5 µL of salmon sperm DNA (AM9680, Thermo Fisher Scientific,), 2.5 µL of pre-cooled 8x IP buffer (80 mM phosphate pH 7.4, 800 mM NaCl, 0.4% Triton X-100), and antibody-coated beads, which were prepared as described as follows. Beads were coated with antibodies by first mixing 1.25 µL of protein G Dynabeads (10003D, Thermo Fisher Scientific) and 1.25 µL anti-rabbit Dynabeads (11203D, Thermo Fisher Scientific). They were then washed twice using 100 µL pre-cooled 1x IP buffer, followed by addition of 5 µL 1x IP buffer, 0.25 µL salmon sperm DNA, 0.5 µL rabbit anti-mouse IgG (ab6709, Abcam, Cambridge, United Kingdom), and 0.5 µL BPDE antibody (BPDE monoclonal antibody (8E11), MA1-40270, Thermo Fisher Scientific).(9) The beads were suspended gently by pipetting to avoid inducing foam, then

rotated at 4 °C overnight on tube revolver (88881001, Thermo Fisher Scientific) with oscillation mode. The antibody-coated beads were washed using 100 µL pre-cooled 1x IP buffer and mixed with the DNA solution described above. The mixture was suspended and rotated for immunoprecipitation at 4 °C overnight on tube revolver with oscillation mode. The beads from immunoprecipitation were washed three times with 200 µL 1x IP buffer and once with 1x TE buffer, then eluted twice with 50 µL pre-warmed elution buffer (10 mM Tris-Cl pH 8.0, 1 mM EDTA, 1% SDS) at 65 °C, 1100 rpm for 5 min. The DNA in combined elution solution was extracted by phenol-chloroform extraction and then precipitated by adding 0.1x volume of 3M sodium acetate, 1 µL GlycoBlue (AM9515, Thermo Fisher Scientific) as co-precipitant, and 2.5x volume of ethanol. The pellet was air dried and resuspended in 6 µL 0.1x TE buffer.

Purified DNA was mixed with 1.5 µL of O3P primer (20 µM) and an equal volume of NEBNext Ultra II Q5 Master Mix (New England Biolabs) and incubated with the following conditions in thermocycler (T100, Bio-Rad Laboratories, Hercules, CA, United States) for polymerase extension: 50 s at 98 °C, 5 min at 65 °C, and hold at 37 °C. To digest the excessive amount of primer, 1.5 µL Exonuclease I (M0293, New England Biolabs) was added to the extension mixture, followed by incubating at 37 °C for 15 min. The mixture was then purified with 0.9 volume of AMPure XP (37 µL, 25 µL MilliQ) and eluted with 20 µL 0.1x TE buffer. Eluent was mixed with 2 µL of SH primer (10 µM), 25 µL 1x B&W buffer (5 mM Tris-HCl pH 8.0, 0.5 mM EDTA, 1 M NaCl, 0.1% Tween20), and subjected to a slow annealing process using thermocycler with the following conditions: 2 min at 98 °C, then cooling at 1 min/°C from 97 °C to 76 °C, 5 min/°C from 75 °C to 55 °C, 1 min/°C from 54 °C to 25 °C, and hold at 4 °C. The annealing product was stored at -20 °C for later processing. Next, 10 µL Dynabeads MyOne Streptavidin C1 (65001, Thermo Fisher Scientific) were washed twice with 1x B&W buffer, resuspended with 5 µL 5x binding buffer (50 mM Tris-HCl, pH 8.0, 5 mM EDTA, 2.5 M NaCl, 0.1% Tween20, 0.1% CA-630, 25 mM $MgCl_2$) and added to the annealing product. The mixture was rotated for 1 h at 4 °C on tube revolver with oscillation mode. The supernatant was transferred to a new 1.5 mL tube. Beads were washed with 50 uL 1x B&W buffer, and combined with the supernatants. The DNA in combined supernatants was purified by ethanol precipitation without adding sodium acetate due to the high salt concentration in the supernatant. The air-dried

pellet was resuspended in 6.5 μL 0.1x TE buffer. The purified DNA was denatured for the following AD2 ligation by heating to 98 °C for 2 min, then immediately placed on ice. Then, 1 μL AD2 (40 μM) and 7.5 uL of Instant Stick Ends Ligase Master Mix (M0370, New England Biolabs) were added. The mixture was kept 16 hat 4 °C and purified with 0.8x volume AMPure XP (40 μL, 35 μL Milli-Q), then eluted with 16 μL 0.1x TE buffer. The eluted DNA was amplified using NEB Next Ultra II Q5 Master Mix with indexing primers for Illumina, the amplified products were purified by 0.9x AMPure XP beads and eluted with 20 μL 0.1x TE buffer. The concentration of eluted DNA was determined using Quantus Fluorometer (Promega, Madison, WI, United States). Each library sample (≧ 15 ng) was pooled and purified again by 0.9x AMPure XP beads to remove residual primer-dimmers, then eluted using 20 μL 10 mM tris buffer (pH 8.0). The mixture was further diluted to desired concentration for sequencing. The pooled libraries were sequenced as 2x151 on an Illumina NovaSeq6000 sequencer (Illumina, San Diego, CA, United States).

RNA-sequencing and data processing

RNA sequencing libraries were prepared using Universal Plus mRNA-Seq library preparation kit with NuQuant (Tecan), starting from 200 ng of RNA with 7 min of enzymatic fragmentation and 17 PCR amplification cycles. Libraries were quantified using NuQuant and normalized to 5 nM. The normalized libraries were pooled into multiplexes, sequenced on Illumina NovaSeq6000 paired-end flow cells with 300 cycles, using sequencing reagent kits. RNA-sequencing reads were aligned to the human genome (version hg38) using STAR aligner. Counting the number of reads per gene was performed using quantMode GeneCounts option from STAR pipeline, based on the Ensembl gene annotation hg38. Read count normalization and differential gene expression were assessed using DEseq2 pipeline with the FDR-adjusted p-value and fold-change cut-offs set to 0.05 and $\log_2(1.5)$, respectively. RNA-sequencing data is available at https://gitlab.ethz.ch/yanjiang/bpde.

Whole-genome bisulfite sequencing and data processing

DNA methylation sequencing libraries were prepared using the Ultralow Methyl-Seq with TrueMethyl oxBS library preparation kit (Tecan), starting from 100 ng DNA fragmented with a sonicator (Covaris E220) and using 7 PCR amplification cycles. Libraries were quantified (Qubit) and normalized to 5 nM. Normalized libraries were pooled into multiplexes sequenced on Illumina

NovaSeq6000 paired-end flow cells with 200 cycle sequencing reagent kits. Sequencing reads were aligned to the human genome (hg38) using the qAlign function in the QuasR package (v 1.10.0) (10), with alignment parameters fitting directional bisulfite-converted libraries. Methylation was quantified using the qMeth function from the QuasR package. Only cytosines in a CpG context were considered, and the counts were strand-combined. Counts were summed per region, and methylation levels corresponding to the ratio between methylated and total events were presented as a 0 to 1 range, with 0 being a fully unmethylated state (methyl counts = 0) and 1 being a fully methylated state (methyl counts = total counts). LMRs (low methylated regions, including non- and partly methylated regions) were identified using methylSeekR package (11) with default parameters. To simplify the analysis, the LMRs from all the samples were combined in unified catalogs (merged LMRs), respectively, whereby elements with more than 60% overlap were merged to reduce redundancy. FMR (fully methylated regions) were regions not being classified as LMR. Methylation levels of the unified sets of LMRs were quantified separately for every replicate and used to compute differential methylation between experimental groups.

N^2-BPDE-dG sequencing data analysis

Data quality was checked using FastQC, and BBMap (12) was used to filter common adaptor contaminations and the reads containing the AD1B sequence: 5′-GAC-TGG-TTC-CAA-TTG-AAA-GTG-CTC-TTC-CGA-TCT-3′. The paired-end reads were mapped to the reference human genome hg38 using Burrows-Wheeler Aligner (13). Reads were not filtered based on their mapping score. Unmapped and duplicated reads were removed using Samtools (14) and Picard. The reads mapped to the ENCODE Blacklist (15) were removed, then the damage sites were extracted and counted in desired bins using custom scripts and bedtools (16). Further analysis was performed using R 4.1.0 and Bioconductor 3.1.2. The analysis window size was evaluated using NGSopwin (17). Scaling factors calculated using “DESeq2” (18) were applied to samples before analysis. Other R packages used for data visualization include ggseqlogo (19) for DNA logos, ggbio (20) for genome-wide karyogram, and ggcorrplot (21) for the heatmap of genomic features correlations. All other figures were plotted using ggplot2 (22). Damage profiling in genomic regions were performed with deepTools (23). DHS (24), transcription factor binding sites (TFBS) (25, 26), and DNA repeat

data were downloaded from UCSC Table Browser. The ChIP-seq data for histone marks and the tXR-seq data for N^2-BPDE-dG were obtained from previously published datasets (27, 28) (Gene Expression Omnibus [GEO] accession no. GSE56053 and GSE97675). Trinucleotide context of the damage sites was extracted using bedtools and custom scripts (available at https://gitlab.ethz.ch/yanjiang/bpde). For the damage and mutational signature analysis, the damage at same genomic positions were only counted once. Samples were pooled by exposure conditions to perform a non-negative matrix factorization to extract damage signatures using the R package MutationalPatterns (29). Cosine similarities were also calculated using the same package. Aside from the analyses of nucleotide enrichment at damage sites and trinucleotide enrichment around damage sites, we considered only those reads that indicated a G at the -1 position.

References

1. E. E. Bessette *et al.*, Screening for DNA adducts by data-dependent constant neutral loss-triple stage mass spectrometry with a linear quadrupole ion trap mass spectrometer. *Anal Chem* **81**, 809-819 (2009).
2. Q. Ruan *et al.*, Quantification of benzo[a]pyrene diol epoxide DNA-adducts by stable isotope dilution liquid chromatography/tandem mass spectrometry. *Rapid Commun Mass Spectrom* **20**, 1369-1380 (2006).
3. P. Pulkrabek, S. Leffler, I. B. Weinstein, D. Grunberger, Conformation of DNA modified with a dihydrodiol epoxide derivative of benzo[a]pyrene. *Biochemistry* **16**, 3127-3132 (1977).
4. F. A. Beland *et al.*, High-performance liquid chromatography electrospray ionization tandem mass spectrometry for the detection and quantitation of benzo[a]pyrene-DNA adducts. *Chem Res Toxicol* **18**, 1306-1315 (2005).
5. N. E. Geacintov *et al.*, Spectroscopic characteristics and site I/site II classification of cis and trans benzo[a]pyrene diolepoxide enantiomer-guanosine adducts in oligonucleotides and polynucleotides. *Carcinogenesis* **12**, 2099-2108 (1991).
6. H. Mu, N. E. Geacintov, J. H. Min, Y. Zhang, S. Broyde, Nucleotide Excision Repair Lesion-Recognition Protein Rad4 Captures a Pre-Flipped Partner Base in a Benzo[a]pyrene-Derived DNA Lesion: How Structure Impacts the Binding Pathway. *Chem Res Toxicol* **30**, 1344-1354 (2017).
7. E. P. Quinlivan, J. F. Gregory, 3rd, DNA digestion to deoxyribonucleoside: a simplified one-step procedure. *Anal Biochem* **373**, 383-385 (2008).
8. J. J. Klaene *et al.*, Tracking matrix effects in the analysis of DNA adducts of polycyclic aromatic hydrocarbons. *J Chromatogr A* **1439**, 112-123 (2016).
9. R. M. Santella, C. D. Lin, W. L. Cleveland, I. B. Weinstein, Monoclonal antibodies to DNA modified by a benzo[a]pyrene diol epoxide. *Carcinogenesis* **5**, 373-377 (1984).
10. D. Gaidatzis, A. Lerch, F. Hahne, M. B. Stadler, QuasR: quantification and annotation of short reads in R. *Bioinformatics* **31**, 1130-1132 (2015).

11. L. Burger, D. Gaidatzis, D. Schübeler, M. B. Stadler, Identification of active regulatory regions from DNA methylation data. *Nucleic Acids Res* **41**, e155-e155 (2013).
12. B. Bushnell, J. Rood, E. Singer, BBMerge – Accurate paired shotgun read merging via overlap. *PLOS ONE* **12**, e0185056 (2017).
13. H. Li, R. Durbin, Fast and accurate short read alignment with Burrows-Wheeler transform. *Bioinformatics (Oxford, England)* **25**, 1754-1760 (2009).
14. P. Danecek *et al.*, Twelve years of SAMtools and BCFtools. *GigaScience* **10** (2021).
15. H. M. Amemiya, A. Kundaje, A. P. Boyle, The ENCODE Blacklist: Identification of Problematic Regions of the Genome. *Sci Rep-Uk* **9**, 9354 (2019).
16. A. R. Quinlan, I. M. Hall, BEDTools: a flexible suite of utilities for comparing genomic features. *Bioinformatics (Oxford, England)* **26**, 841-842 (2010).
17. A. Gusnanto *et al.*, Estimating optimal window size for analysis of low-coverage next-generation sequence data. *Bioinformatics* **30**, 1823-1829 (2014).
18. M. I. Love, W. Huber, S. Anders, Moderated estimation of fold change and dispersion for RNA-seq data with DESeq2. *Genome Biology* **15**, 550 (2014).
19. O. Wagih, ggseqlogo: a versatile R package for drawing sequence logos. *Bioinformatics (Oxford, England)* **33**, 3645-3647 (2017).
20. T. Yin, D. Cook, M. Lawrence, ggbio: an R package for extending the grammar of graphics for genomic data. *Genome Biology* **13**, R77 (2012).
21. A. Kassambara, ggcorrplot: Visualization of a Correlation Matrix using 'ggplot2'. R package version 0.1.3. (2019).
22. H. Wickham, ggplot2: Elegant Graphics for Data Analysis. *Springer-Verlag New York* https://doi.org/10.1007/978-3-319-24277-4 (2016).
23. F. Ramírez *et al.*, deepTools2: a next generation web server for deep-sequencing data analysis. *Nucleic Acids Res* **44**, W160-W165 (2016).
24. R. E. Thurman *et al.*, The accessible chromatin landscape of the human genome. *Nature* **489**, 75-82 (2012).
25. E. P. Consortium, An integrated encyclopedia of DNA elements in the human genome. *Nature* **489**, 57-74 (2012).
26. C. A. Davis *et al.*, The Encyclopedia of DNA elements (ENCODE): data portal update. *Nucleic Acids Research* **46**, D794-D801 (2017).
27. W. Li *et al.*, Human genome-wide repair map of DNA damage caused by the cigarette smoke carcinogen benzo[a]pyrene. *Proceedings of the National Academy of Sciences* **114**, 6752-6757 (2017).
28. C. C. Jose *et al.*, Nickel exposure induces persistent mesenchymal phenotype in human lung epithelial cells through epigenetic activation of ZEB1. *Mol Carcinogen* **57**, 794-806 (2018).
29. F. Blokzijl, R. Janssen, R. van Boxtel, E. Cuppen, MutationalPatterns: comprehensive genome-wide analysis of mutational processes. *Genome Medicine* **10**, 33 (2018).
30. F. Feng, X. Wang, H. Yuan, H. Wang, Ultra-performance liquid chromatography-tandem mass spectrometry for rapid and highly sensitive analysis of stereoisomers of benzo[a]pyrene diol epoxide-DNA adducts. *J Chromatogr B Analyt Technol Biomed Life Sci* **877**, 2104-2112 (2009).

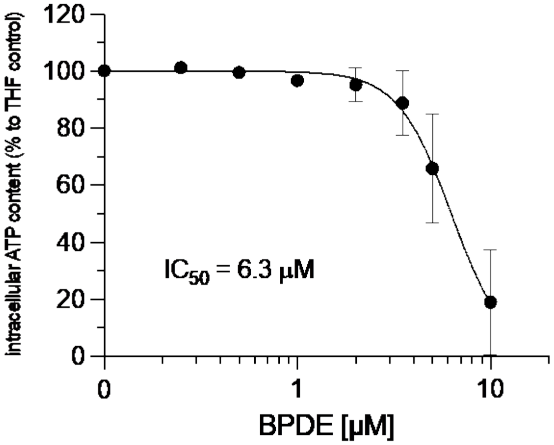

Fig. S1. Cell viability assessed by measuring intracellular ATP content. BEAS-2B cells were exposed to increasing concentrations of BPDE for 24 h. Data represent the mean of four biological replicates +/- SD. The curve is a non-linear regression fit.

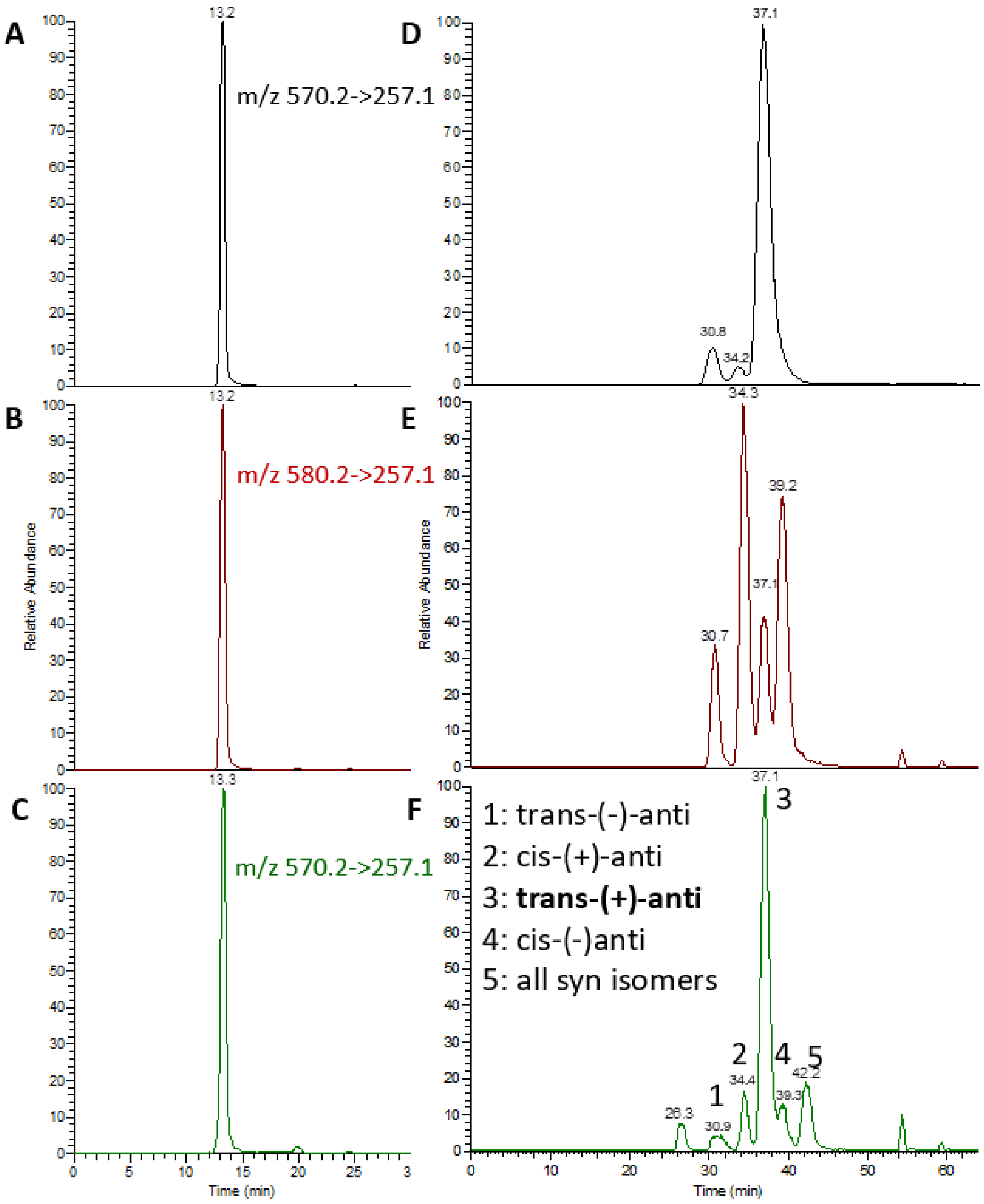

Fig. S2. LC-MS/MS chromatograms of (A) N^2-BPDE-dG DNA adducts from BEAS-2B cells exposed to 2u M BPDE (B) internal standard $^{13}C_{10}$-N^2-BPDE-dG and (C) N^2-BPDE-dG DNA adducts from BEAS-2B cells treated with 20 μM BaP + 10 nM TCDD for 24 h, analyzed with the standard HPLC method that does not separate isomers. (D)-(F) LC-MS/MS chromatograms with separation of isomers (isocratic method) corresponding to (A)-(C). The isomers assignments are made based on previous identification (30).

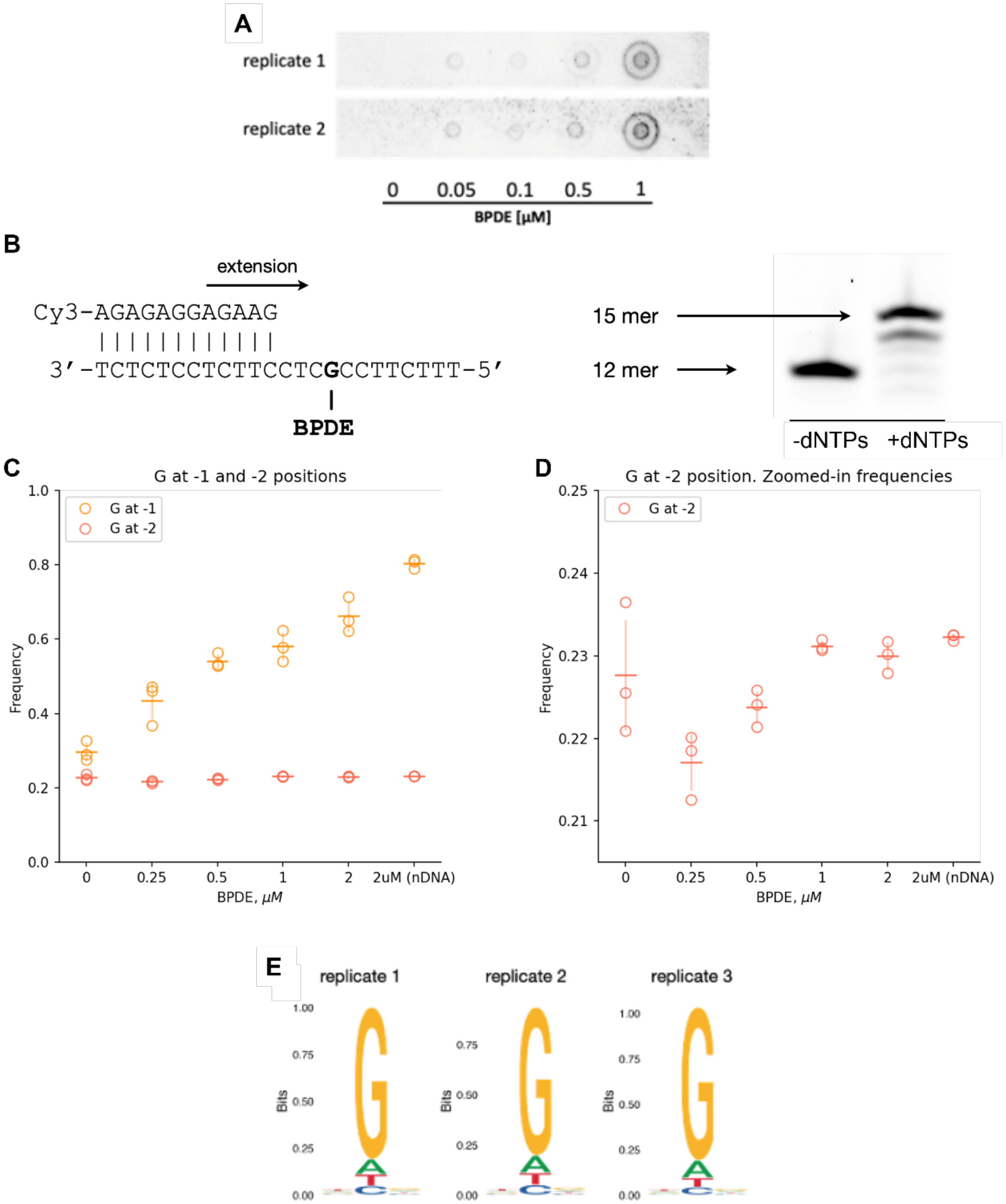

Fig. S3. (A) Antibody binding capacity tested using a dot blot assay. Inputs were nDNA reacted with increasing concentration of BPDE using the same method described in the materials and methods section. Loading amount is 0.5 µg. (B) Q5 polymerase extension experiment and corresponding gel electrophoresis results. (C-D) The frequencies of G in the -1 and -2 positions (relative to the 5’ end of sequencing reads) across exposure conditions. Circles are replicates, horizontal dashes are mean values, and vertical lines are mean values ± std. (E) Trinucleotide logo from BPDE-exposed nDNA indicating the context of the damage site.

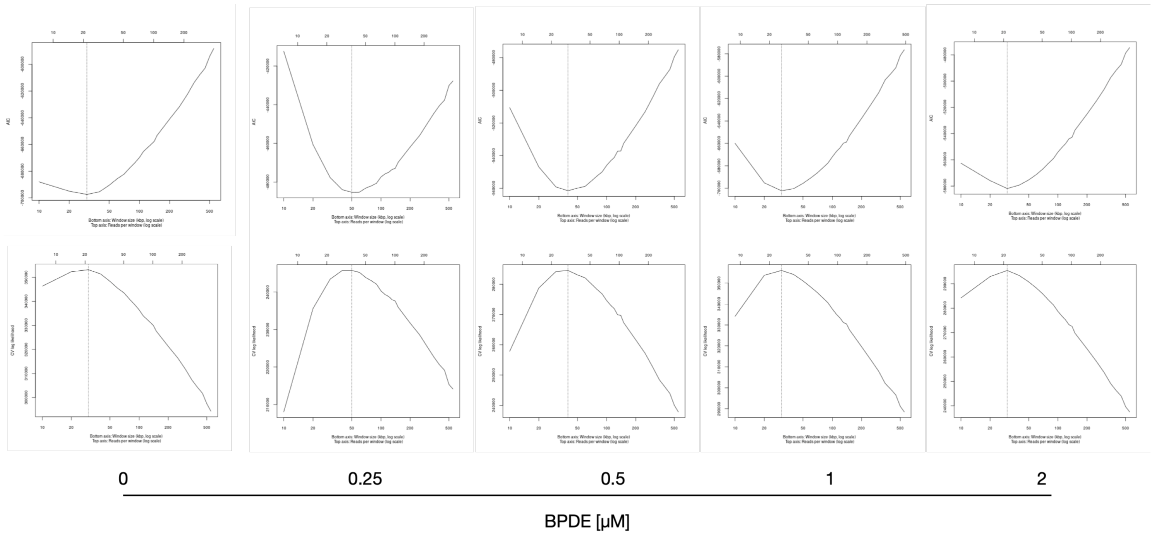

Fig. S4. Representative results of Akaike's information criterion (AIC, top row) and cross-validation (CV) log-likelihood (bottom row), as a function of different window sizes (bottom axis in each figure) or the corresponding number of reads per window (top axis in each figure) in negative control and BPDE exposed cells.

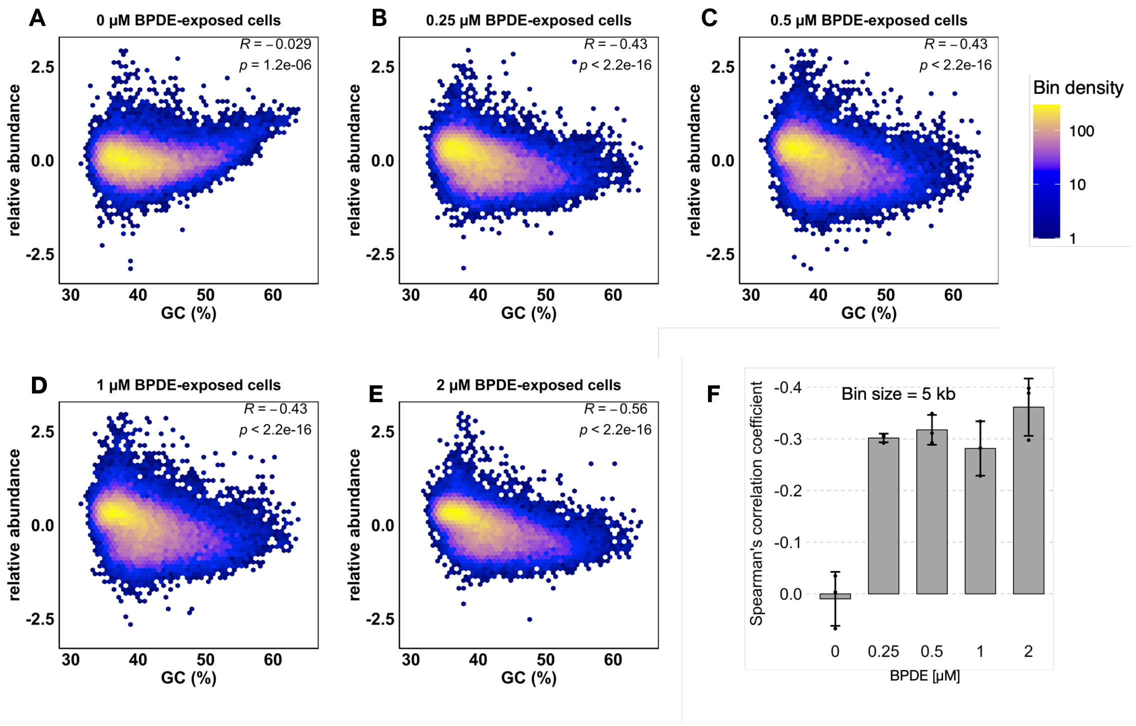

Fig. S5. Relative abundance of N^2-BPDE-dG from negative control and BPDE-exposed cells along GC content. (A-E) Scatter plots showing the distribution of relative abundance from negative control and BPDE-exposed cells along GC content. Spearman's correlation coefficients are shown at the top right of each plot. Data represent the mean of three biological replicates. (F) Bar plot showing the correlation between the relative abundance of N^2-BPDE-dG with GC-content in all cell samples. Data show individual values of three biological replicates +/- SD calculated from 5 kb bins.

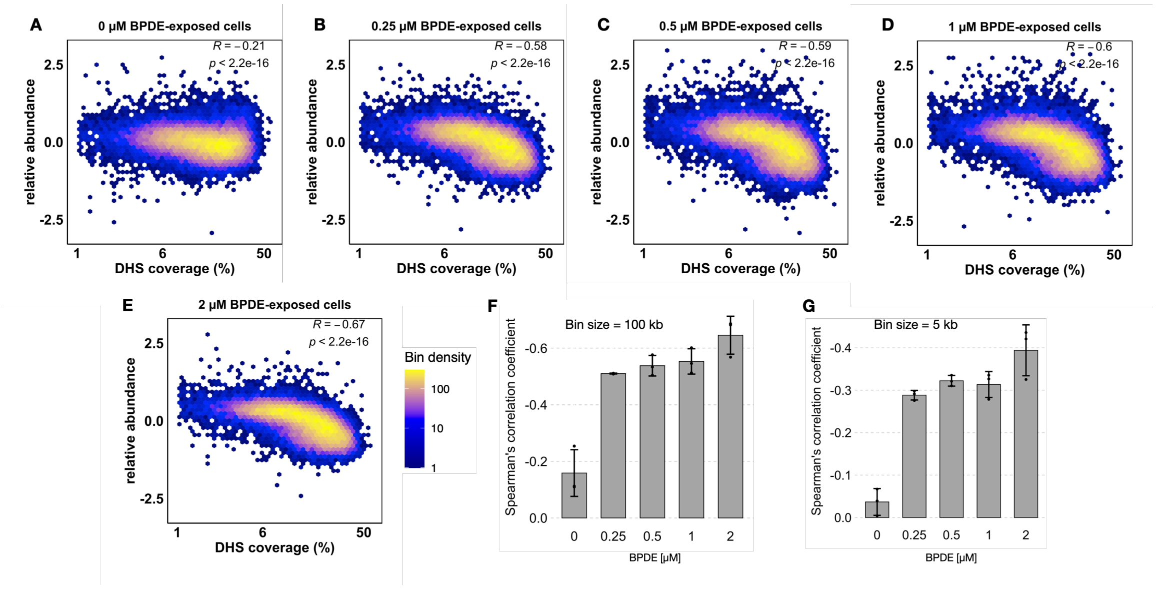

Fig. S6. Relative abundance of N^2-BPDE-dG from negative control and BPDE-exposed cells along DHS coverage. (A-E) Scatter plots showing the distribution of relative abundance from negative control and BPDE-exposed cells along DHS coverage. Spearman's correlation coefficients are shown at the top right of each plot. Data represent the mean of three biological replicates. (F, G) Bar plots showing the correlation between the relative abundance of N^2-BPDE-dG with DHS coverage in all cell samples. Data show individual values of three biological replicates +/- SD calculated from 100 and 5 kb bins, respectively.

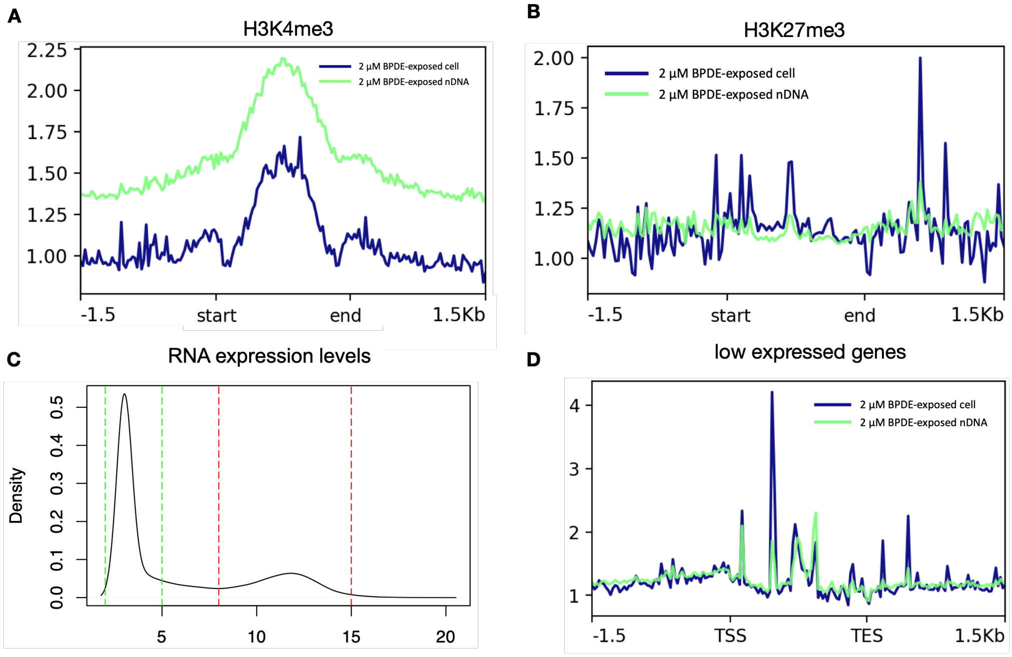

Fig. S7. (A, B) Damage profile in H3K4me3 and H3K24me3 regions from 2 μM BPDE-exposed cells and nDNA. Data represents the mean calculated from three replicates in 25 bp bins. (C) Density plot showing a distribution of representative RNA expression levels in cell samples. Low expressed genes are defined as the expression level < 3, while high expressed genes are defined as the genes with expression level between 8 and 15. (D) Damage profile from 2 μM BPDE-exposed cells and nDNA in low (n = 22,645) expressed gene regions. Data represent the mean calculated from three replicates in a bin size of 25 bp.

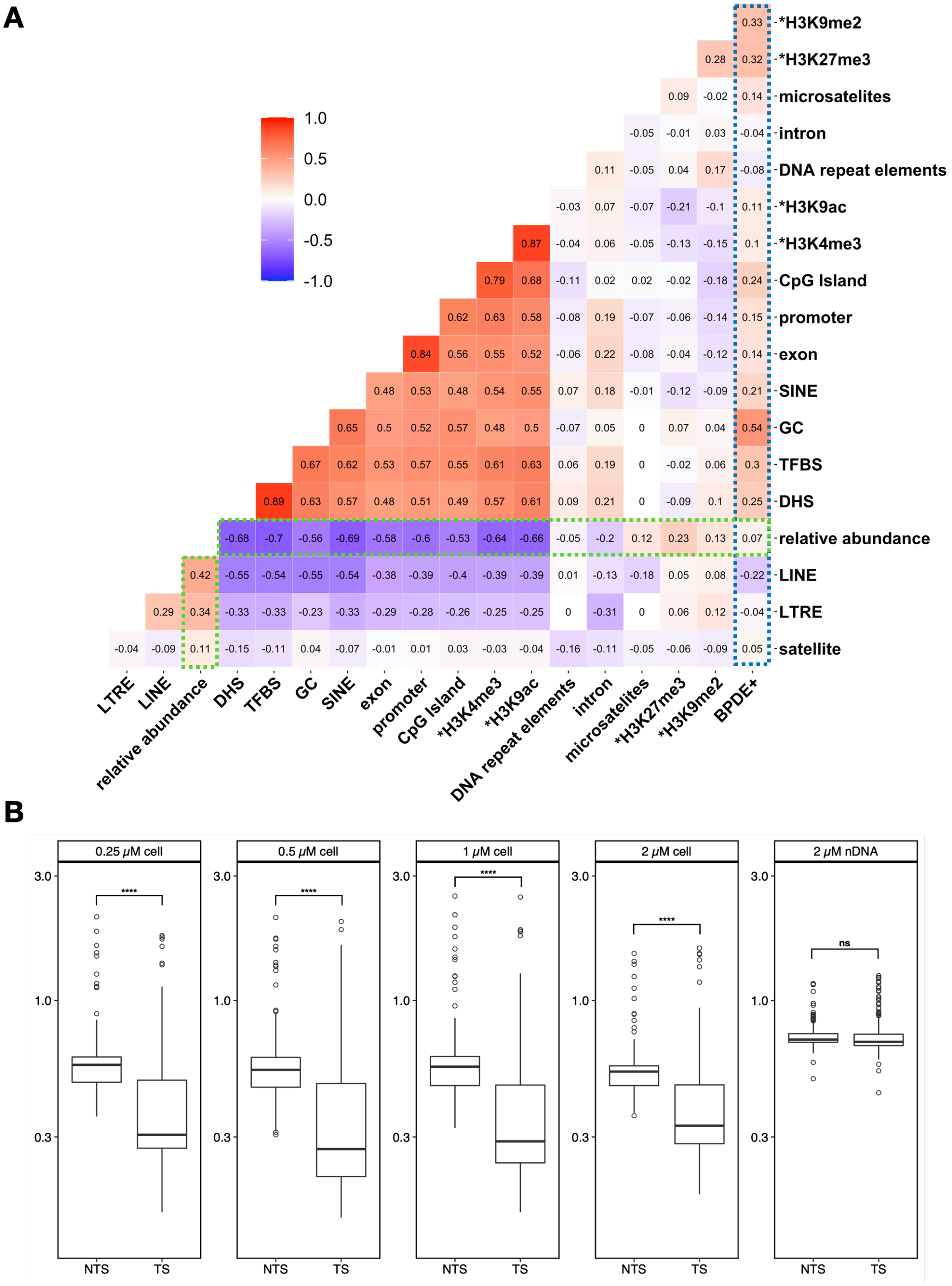

Fig. S8. (A) Heat-map representing the correlation between variables. Variables including coverage of genomic features per bin, BPDE+ (site count of 2 uM exposed cell, highlighted by blue dash lines), and relative abundance (2 uM BPDE-exposed cells, highlighted by green dash lines). (B) Boxplot showing the damage distribution on non-transcribed strand (NTS) and transcribed strand (TS) of high expressed genes (n= 10,612) from all exposed samples. Data represent the mean calculated from three replicates in a bin size of 25 bp. The significant test was calculated using paired t-test.

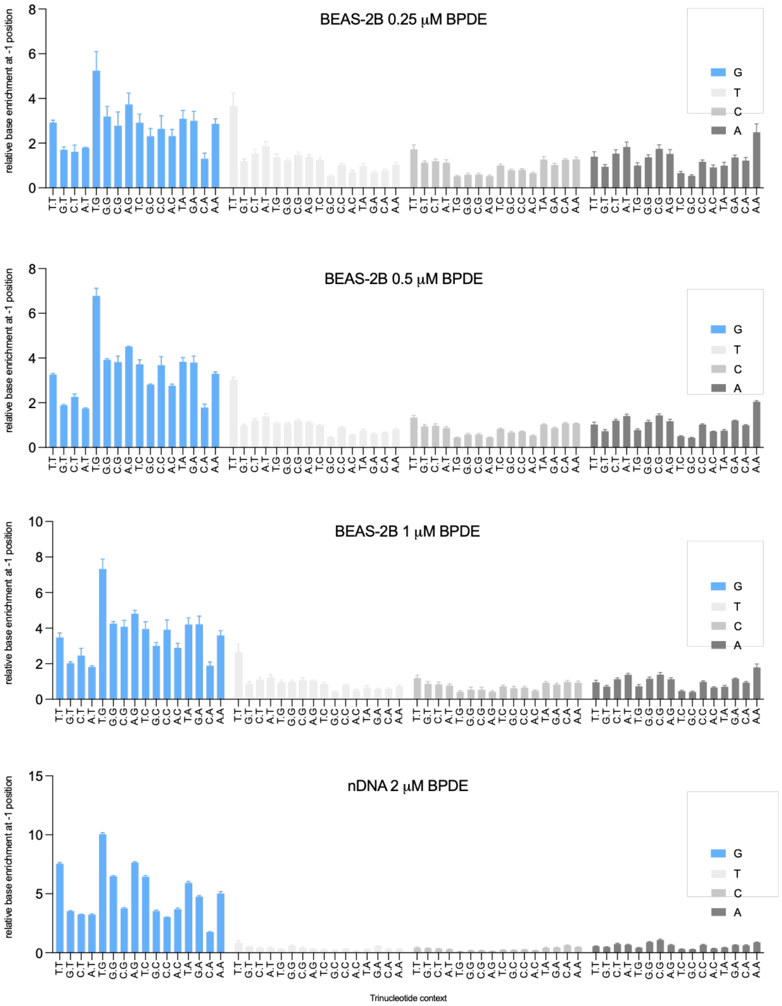

Fig. S9. Relative enrichment of DNA bases at -1 position of the sequencing read start in BEAS-2B cells exposed to 0.25, 0.5 and 1 μM (±)-anti-BPDE and in nDNA exposed to 2 μM (±)-anti-BPDE

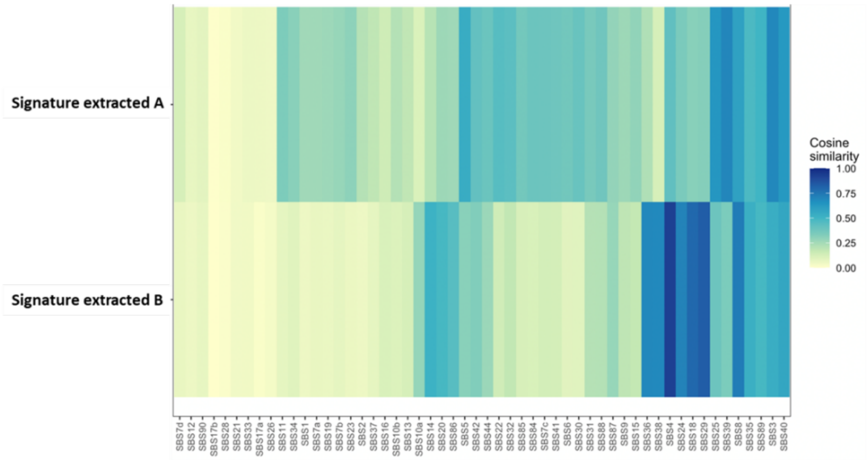

Fig. S10. Cosine similarities between extracted DNA damage signatures, A and B, and COSMIC human cancer mutational signatures.

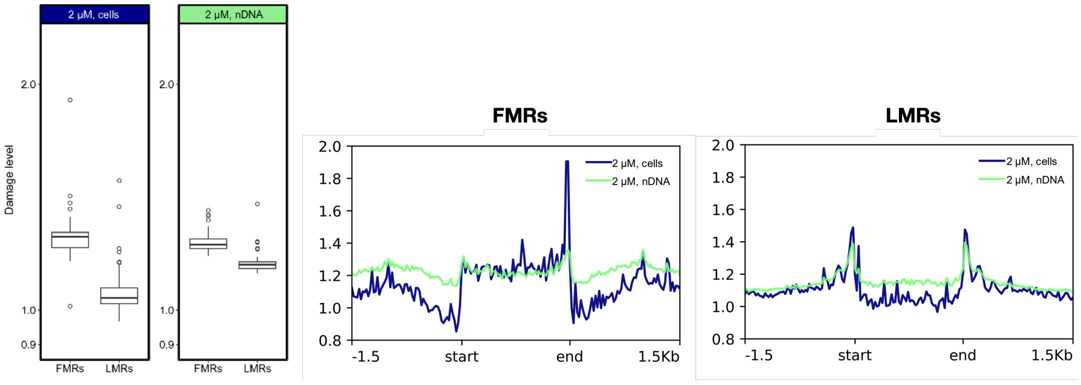

Fig. S11. Comparing damage levels between DNA regions with different methylation status probed in bisulfite sequencing. Regions of the genome were categorized as LMR, i.e. low methylated regions, including non- and partly methylated regions, or FMRs, i.e. fully methylated regions.

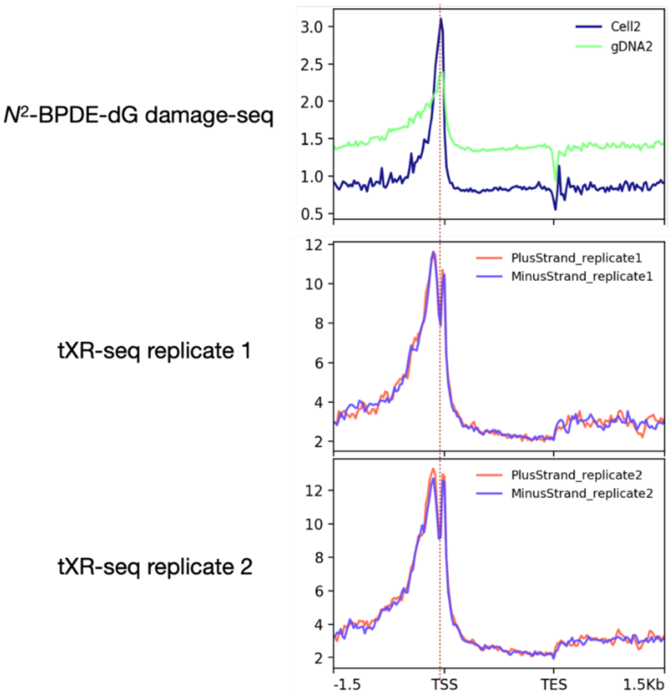

Fig. S12. Comparison of the N2-BPDE-dG distribution with NER levels from published tXR-seq data.

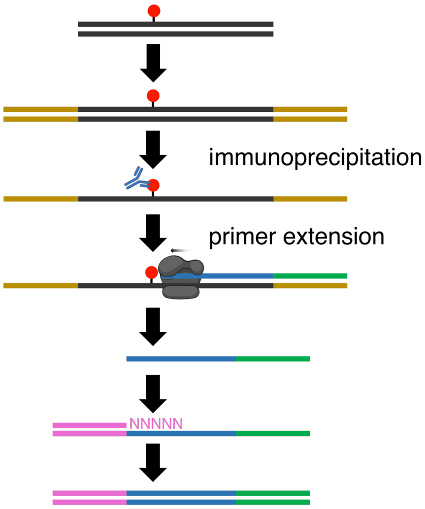

Fig. S13. Schematic of BPDE-dG damage sequencing

Table S1. Transitions and collision energies used to quantify N^2-BPDE-dG by LC-MS/MS.

Analyte	parent mass (m/z)	fragment mass (m/z)	collision energy (V)
N^2-BPDE-dG	570.2	257.1	30
		285.1	20
		454.1	10
$^{13}C_{10}$- N^2-BPDE-dG	580.2	257.1	30
		285.1	20
		459.1	10

Table S2. Sequences of oligonucleotides.

Name	Sequence
Extension primer	Cy3-AGAGAGGAGAAG
Extension template	5'-TTTCTTCC**G**CTCCTTCTCCTCTCT
AD1T	5'-phos-GATCGGAAGAGCACACGTCTGAACTCCAGTCA-SpC3
AD1B	5'-NNNNNGACTGGTTCCAATTGAAAGTGCTCTTCCGATC*T
AD2T	5'-phos-AGATCGGAAGAGCGTCGTGTAGGGAAAGAGTGT-SpC3
AD2B	5'-ACACTCTTTCCCTACACGACGCTCTTCCGATCTNNNNN-SpC3
O3P	5'-GACTGGAGTTCAGACGTGTGCTCTTCCGATCT
SH	5'-biotin-NNGACTGGTTCCAATTGAAAGTGCTCTTCCG-SpC3

Note: **G** represents N^2-BPDE-dG, "*" represent phosphorothioate bond
